# Gating and ion selectivity of Channelrhodopsins are critical for photo-activated orientation of Chlamydomonas as shown by in vivo point mutation

**DOI:** 10.1038/s41467-022-35018-6

**Published:** 2022-11-25

**Authors:** Olga Baidukova, Johannes Oppermann, Simon Kelterborn, Rodrigo G. Fernandez Lahore, Dimitri Schumacher, Heide Evers, Yousef Yari Kamrani, Peter Hegemann

**Affiliations:** 1 Institute of Biology, Experimental Biophysics, Humboldt University of Berlin, Berlin, Germany; 2 Institute for Vegetative Physiology, Charité - Universitätsmedizin, Berlin, Germany

**Keywords:** Cellular motility, Permeation and transport, Membrane proteins, Voltage clamp

## Abstract

The green unicellular alga *Chlamydomonas reinhardtii* with two photoreceptors called channelrhodopsins is a model organism that gave birth to a new scientific field of biomedical studies, optogenetics. Although channelrhodopsins are helping to decipher the activity of the human brain, their functionality has never been extensively studied in the organism of origin, mainly due to the difficulties connected to reverse genetic interventions. In this study, we present a CRISPR-Cas9-based technique that enables a precise in vivo exchange of single amino acids in a selected gene. To shed light on the function of channelrhodopsins ChR1 (C1) and ChR2 (C2) in vivo, we deleted both channelrhodopsins independently in the wild-type strain and introduced point mutations in the remaining channel, causing modified photocycle kinetics and ion selectivity. The mutated strains, ΔC1C2-E123T, ΔC1C2-E90R and ΔC1C2-E90Q, showed about 100-fold decrease in photosensitivity, a reduced photophobic response and faster light adaptation rates due to accelerated photocycle kinetics and reduced Ca^2+^ conductance. Moreover, the ΔC1C2-E90Q with an additionally reduced H^+^ permeability produced an electrical response only in the presence of Na^+^ ions, highlighting a contribution and importance of H^+^ conductance to photocurrents in the wild-type algae. Finally, in the ΔC1C2-E90R strain with the channelrhodopsin selectivity converted to anions, no photo-responses were detected. We conclude that the precise photocycle kinetics and the particular ion selectivity of channelrhodopsins are the key parameters for efficient phototaxis in low light conditions.

## Abstract (web summary)

Channelrhodopsin photoreceptors are responsible for phototaxis in C. reinhardtii. Here, authors introduce point mutations in the channel which modify photocycle kinetics and ion selectivity, resulting in a deeper understanding of phototaxis in low light conditions.

## Introduction

The biflagellate unicellular green alga *Chlamydomonas reinhardtii* is a widely used model organism for fundamental research. Its swims forward in a breaststroke style with a helical motion of 2 Hz, and during illumination, they move to or away from a light source, a phenomenon called phototaxis. Upon application of high-intensity light flashes, they perform a photophobic response with backward swimming for about a second and a new orientation thereafter^1^. Channelrhodopsins ChR1 and ChR2 (C1 and C2, respectively) are photoreceptor proteins responsible for this light-induced behaviour of the algae^2^. With these photoreceptors located in the part of the plasma membrane overlaying the eyespot and the optical system of carotenoid layers (named functional eye in the following), cells can detect the direction of the light source in order to populate areas with optimal conditions for photosynthesis^3,4^. Channelrhodopsins function as ion channels directly gated by light that upon photon absorption promote fast proton and cation inward currents resulting in plasma membrane depolarization^5,6^. Upon flash stimulation with photon exposures between 5 and 100% photocurrent saturation, flash-induced photocurrents in Chlamydomonas rise without any detectable delay (τ <50 µs), peak after 1–2 ms and decay within 20 ms depending on the light intensity^2,7,8^. The short delay has been interpreted as a direct coupling of rhodopsin and ion channel in a photoreceptor-ion channel complex^7,9^. At low light intensities (below 5 × 10^18^ photons/m^2^s) when less than 5 % of rhodopsins are excited, the photocurrent is delayed for several milliseconds, suggesting that unidentified voltage-gated Ca^2+^ channels with low abundance are involved and amplify the electrical signal^8,10^. The amplification may reach a factor of 1000 near the threshold when single photons are detected as it was originally suggested for *Haematococcus pluvialis* photocurrents^8,10,11^. When the depolarization of the plasma membrane, resulting from the photoreceptor current activation, reaches a certain threshold^7^, it leads to the opening of voltage-gated Ca^2+^ channels CAV2 and initiation of flagellar currents^12^. These currents trigger switching from forward to reverse swimming (photophobic response) preceded by an alteration of beating mode in the *trans* flagellum^13,14^. At low light intensities, no flagellar currents are detected^7^ and the amplitude of the flagellar beating pattern changes only slightly without loss of synchrony in beating mode. Moreover, with rising photon exposure Chlamydomonas photoreceptor currents increase in amplitude and decay faster due to faster membrane depolarization^7,14^. Further, electrical measurements suggest that under physiological conditions the photoreceptor currents are carried mainly by Ca^2+^ because the currents almost completely disappear after adding the Ca^2+^ chelator BAPTA or L-type Ca^2+^ channel inhibitors verapamil and pimozide^14^. However, small photoreceptor currents have still been detectable in the absence of Ca^2+^, implying that protons and other monovalent cations are conducted^7,15^.

**Fig. 1:**
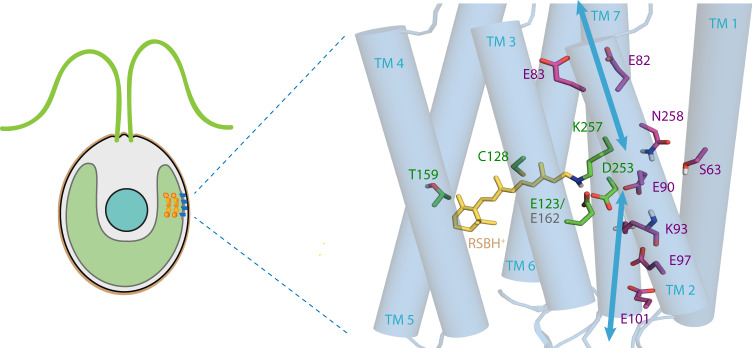
The crystal structure of ChR2 from *Chlamydomonas reinhardtii* (PDB: 6EID^58^). Protonated retinal Schiff base (RSBH^+^) is depicted in yellow. The key residues of ion-conducting pore (magenta) and retinal binding pocket (green) with protonation states for polar residues are indicated by sticks (oxygen in red, nitrogen in blue, polar protons in white). Blue arrows show the ion permeation pathway.

Up until now, photocycle kinetics of the channel and its ion selectivity, the characteristics of channelrhodopsins that underlie phototactic and photophobic responses of Chlamydomonas, have been extensively studied in vitro in search of improved optogenetic tools^16^. According to these findings, the off-kinetics of ChR1 are twice as fast as in ChR2 due to a reduced lifetime of the conducting state^17^. However, data on heterologous expressed wildtype or mutant ChR1 is sparse due to low expression levels^5,18^. In search of modulation mechanisms of photocycle kinetics, it was shown that mutation of ChR2-E123 in the retinal binding pocket to threonine (ChETA: Channelrhodopsin-ET-accelerated) leads to an accelerated and an almost voltage-independent photocycle with reduced photocurrent amplitudes^19^ (Fig. 1). The homologous mutation E162T in ChR1 is expected to perform similarly^20^. Furthermore, the central gate residue E90 in ChR2 is the key element of the channel ion selectivity and a regulator of the ion permeation pathway. Its replacement by glutamine strongly reduces H^+^ conductance due to the inability of E90Q to bind H^+^ and abolishes late photocurrents during light adaptation^21,22^. E90R, on the other hand, inverts the channel from a cation-selective to an anion-selective channel with negligible Na^+^ or Ca^2+^ permeability^23^. To study different ChR selectivities in vivo and to overcome the strong genomic positional effects in *Chlamydomonas reinhardtii* causing dysregulation of gene expression, we have introduced point mutations in the native gene instead of replacing the whole ChR genes with modified gene homologs. To achieve these goals, we improved our Cas9-based gene editing technique for Chlamydomonas that allow for not only inactivation of selected genes but also the ability to introduce specific minor changes, such as an exchange of single nucleotides.

## Results

### Design of point mutations in channelrhodopsin genes

For CRISPR-Cas9-based editing of the Chlamydomonas genome, we used technique in which recombinant *Streptococcus pyogenes* Cas9 is pre-assembled with synthetic guide RNA (gRNA) and transformed directly into the cells as ribonucleoprotein (RNP) complexes^24,25^. The Cas9 RNP complex induces double-stranded DNA breaks that are mostly repaired by error-prone non-homologous end-joining or the more rarely occurring homology-directed repair (HDR) mechanisms that use homologous DNA as repair templates. Precise single-base pair exchanges depend on the HDR mechanism. One technical challenge in introducing single base-pair mutations is the identification of cells that resulted from such rare repair events.

**Fig. 2:**
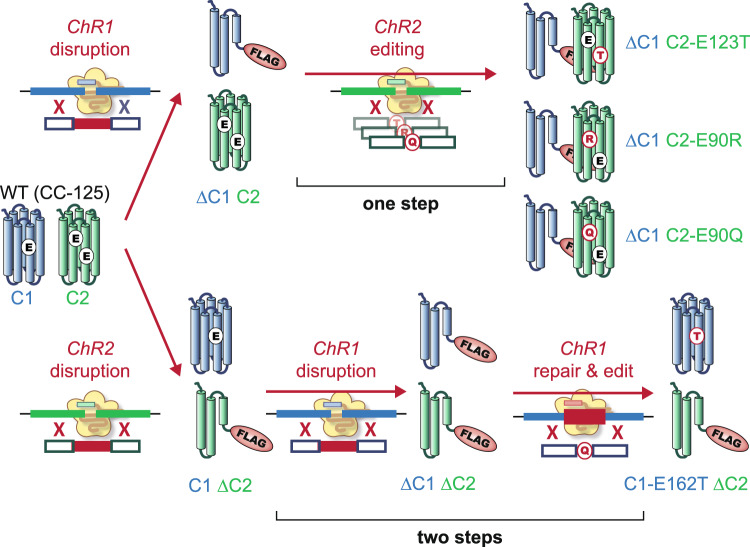
Two approaches for deletion and target-specific point mutation in ChR genes using the CRISPR-Cas9 system and homology-directed repair. The ribonucleoprotein (RNP) complex consisting of a site-oriented guide RNA and a protein nuclease Cas9 cleaves genomic DNA and creates double-stranded DNA breaks that can be sealed via homology-directed repair in one or two steps. (1) The one-step approach enables a simultaneous repair of the break via recombination between the genomic DNA and a homologous donor DNA with an encoded point mutation. (2) The two-step approach requires, at first, an insertion of a specific short FLAG sequence and inactivation of the gene that can be repaired in the next step via homologous recombination with a donor DNA encoding a point mutation.

We applied two strategies to generate ChR mutants with the desired point mutations. Overall, both approaches used co-transformation of the Cas9 RNP complex with a plasmid carrying an antibiotic-resistance marker, followed by selection on plates for cells with antibiotic resistance. Then, the first approach directly integrated the point mutation and all potential clones were sequenced without pre-screening. As a second approach we propose a two-step transformation approach that allowed the identification of correct clones via change in the size of the PCR product (Fig. 2). Here, we first integrated a unique synthetic 29–31 bp DNA sequence at the target site which we termed “FLAG”-sequence. The FLAG sequence includes stop codons, leading to termination of translation, and a Cas9 target site, allowing a second transformation to remove the FLAG-sequence and thereby introducing the base-pair exchange. The introduction or removal of the FLAG sequence with two subsequent transformations is more time-consuming and two antibiotic selection markers are required but each step can be screened for changes in the target locus size with a low-cost standard PCR. In addition, the first step of the two-step transformation approach could be hastened by screening phenotypically for a gene knock-out, e. g. using a lack of growth on acetate in case of genes involved in photosynthesis^26^.

The RNP complex induces double-stranded DNA breaks that can be repaired by non-homologous end-joining or via recombination with a homologous DNA template (Fig. 2). Parallel to the DNA repair by recombination, minor targeted exchanges of single nucleotides were introduced in one or two steps in some cells. The one-step approach recruited direct recombination with a homologous DNA template that included a point mutation of interest. The two-step approach first required a deactivation of the target gene by an insertion of a known 30 bp sequence called FLAG and then the subsequent repair of the gene with a simultaneous exchange of single amino acids. On one hand, the FLAG insertion and its loss via homologous recombination has the advantage of facilitating the detection of a repaired gene with a single nucleotide exchange due to the size difference of amplified regions, including target sites. On the other hand, the two-step approach was more time-consuming and required the use of two antibiotic selection markers for sorting out positive colonies.

**Fig. 3:**
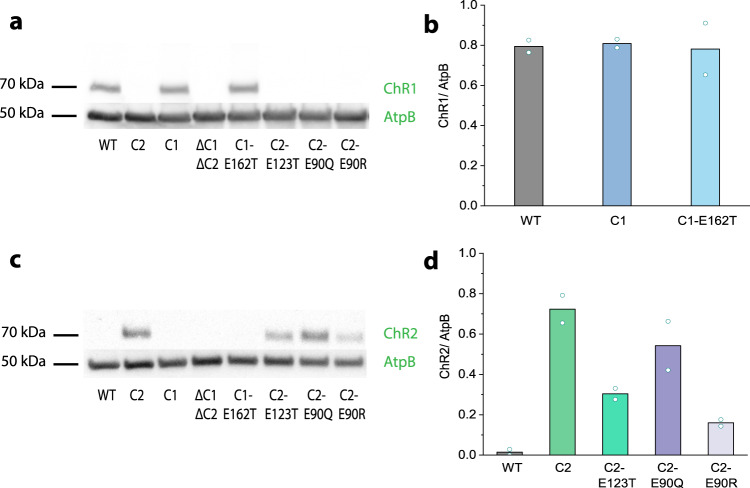
Immunoblot evaluation of ChR expression levels in *Chlamydomonas reinhardtii*. **a** Protein immunoblotting of wild-type gamete cells, C1 and C2 strains, the double knock-out ΔC1ΔC2 and point mutants using anti-ChR1 antiserum and secondary HRP-conjugated antibody for chemiluminescence detection. An ATP synthase subunit B (AtpB) antibody (53 kDa) was used as a loading control. **b** ChR1 expression levels relative to the loading control AtpB. **c** Protein immunoblotting of the wild-type gamete cells, C1 and C2 strains, the double knock-out ΔC1ΔC2 and mutants using anti-ChR2 antiserum and secondary HRP-conjugated antibody for chemiluminescence detection. **d** ChR2 expression levels relative to AtpB. Lines represent the mean value. *N* = 2 independent experiments. Source data are available as a Source Data file.

To exclude possible off-target effects in the Chlamydomonas genome that may influence the behavioural performance of the mutant strains, we studied two independent clones for each point mutation. In addition, to have a clear background for a single nucleotide exchange in one channelrhodopsin, we used parent strains ΔC1 and ΔC2 where the other channelrhodopsins were still active (C2 and C1 in the following). We ran our experiments on the wild-type CC125 cells because after ChR1 deletion ChR2 becomes strongly upregulated in this strain (Fig. 3). Only this upregulation, which was not found in the strains CC3403^24^ or CW2^18^, allowed us to functionally study ChR2.

The mutants ΔC1C2-E123T, ΔC1C2-E90R and ΔC1C2-E90Q, named just C2-E123T, C2-E90R and C2-E90Q in the following, were obtained using the straightforward one-step approach. The ΔC2C1-E162T mutant, named C1-E162T in the following, underwent a two-step transformation (Fig. 2). Overall, the straightforward one-step approach has been proven to be less time-consuming and equally efficient for finding enough correct mutations. For point mutation of the gene in two steps, the insertion of the FLAG sequence needed to be precise as well, which was only the case in 1–2 % of the selected clones.

The disruption efficiency of genes using homology-directed repair is 5 − 15 %^24^ while precise gene-editing events needed for single point mutations were roughly 1 % for the optimized one-step procedure. However, several limitations made a precise single amino acid exchange challenging. First, the selected position for a point mutation of interest was a limiting factor for the choice of an efficient target-specific gRNA because the success of the precise editing event depends on how strongly a gRNA is bound to and interacts with a target DNA. Second, the success of homologous recombination relied on a close proximity of the mutation site to the site of the double-stranded DNA break, which should not exceed a distance of more than 10–20 bp^27^. Finally, a GC-rich environment of the target site^28,29^ as well as the inaccessibility of chromatin for gRNA binding^30^ can limit error-free homologous recombination. These factors may explain a different efficiency rate of precise editing events for C1-E162T, C2-E90Q and C2-E90R mutants, which was around 1 % while it was below 1 % for the ChETA mutation C2-E123T.

After the mutant strains were confirmed by sequencing (Supplement Figs. 1, 2), we evaluated the expression levels of ChR1 and ChR2 by protein SDS-PAGE and immunoblotting. Interestingly, while the wild-type lacked ChR2, with no more than 2% present, the ChR2 protein expression levels were highly up-regulated upon ChR1 inactivation. In contrast, the relative expression levels of ChR1 in the wild-type (WT), in the strain with only ChR1 present (C1) and in the ChETA mutant C1-E162T were all detected as bands at approximately 70 kDa with very similar intensity (Fig. 3a, b). In line with these differences, ChR2 expression observed at 70 kDa was reduced for the point mutants when compared to the strain with intact ChR2 (C2), i. e., 75 % for C2-E90Q, 42 % for C2-E123T and 22 % for C2-E90R (Fig. 3c, d). Finally, in the mutant with both ChR1 and ChR2 inactivated (ΔC1ΔC2), no immunoreactive protein was detectable.

### Evaluation of phototactic response

To unravel the channelrhodopsin properties that are critical for phototactic performance and photosensitivity in Chlamydomonas, we applied a light scattering technique that monitors the photoorientation of cells over time. Upon light exposure, the light scattered from the cells changes its intensity during cell reorientation^31^. The relative photosensitivity was determined as a minimal irradiance needed for phototaxis from the curves of directional change plotted over light intensity and expressed in percent relative to the reference value (3.0 × 10^15^ photons·m^−2^s^−1^ for WT).

**Fig. 4:**
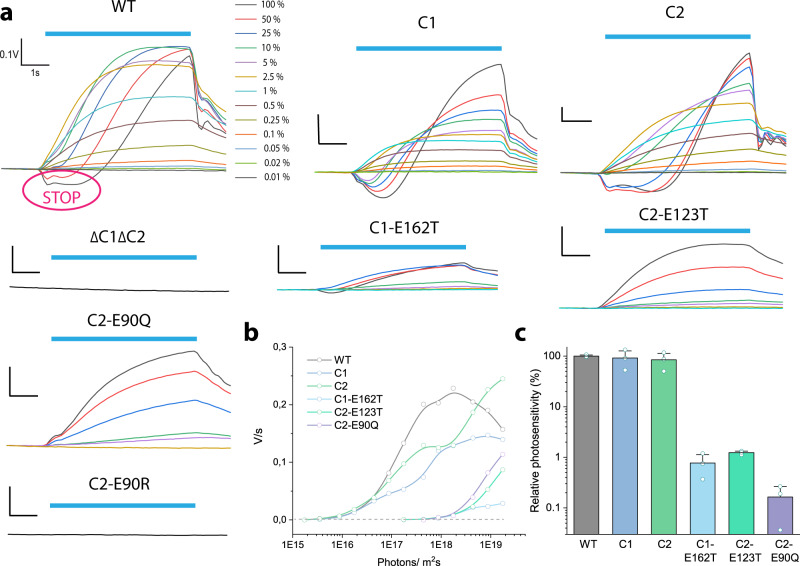
Phototactic response of *Chlamydomonas reinhardtii*. **a** Change of orientation in a suspension of 1 × 10^6^ cells/ml containing the wild-type, C1 and C2 strains, the double knock-out ΔC1ΔC2 and point mutants. The cells were illuminated with 470-nm light for 6 s (blue bar) perpendicular to the monitoring light. 100 % light intensity corresponds to 1.75 × 10^19^ photons·m^−2^s^−1^. The photophobic response is marked as STOP. **b** Rate of directional change is plotted versus light intensity. **c** Photosensitivity normalized to the mean irradiance value (3.05 × 10^15^ photons·m^-2^s^-1^) that wildtype cells needed to perform phototaxis. Lines and bars represent the mean value ± S.E. *N* = 3 independent experiments. Source data are available as a Source Data file.

Dark-adapted wild-type cells, C1 and C2 strains reacted to 470nm-light pulses already at > 0.02 % of the maximal light intensity (3.5 × 10^15^ photons/m^2^s) (Fig. 4a, b). The double knock-out ΔC1ΔC2 did not show any phototaxis. This confirms that, first, ChR1 and ChR2 are both photoreceptors mediating phototaxis in Chlamydomonas and second, the drastic upregulation of ChR2 upon deletion of ChR1 rescues the phototactic behavior in ChR1-deprived cells.

At light intensities of 1 to 10%, the signal saturates fast (<2 s) in the C1 strain indicating reorientation of the cell movement and the amplitudes are reduced compared to wild-type cells. The C2 cells need longer to reorient themselves. The delay of 1–2 s of the signal increase seen at high intensities after the illumination start is defined as a photophobic response (STOP). The phobic response in the C2 cells is even more pronounced compared to wild-type cells, which can be explained by the larger Ca^2+^ conductance of C2 according to our electrophysiological study in the neuroblastoma ND7/23 cells (shown below) and the previous study in *Xenopus* oocytes^17^. A more dramatic change was observed for the strains with disrupted C1 and a single point mutation in this channel, namely C1-E162T, C2-E123T and C2-E90Q, where a nearly 100-times higher irradiance was needed to invoke a phototactic reaction (Fig. 4a, b).

Finally, the chloride conducting C2-E90R showed a complete lack of photoresponse in the alga. The reduced levels of ChR2 expression in the mutant play only a minor role, since the sensitivity is proportional to the photoreceptor molecules in the eye and a reduction to 20% should only account for a sensitivity reduction of fivefold^7^. However, changes in retinal affinity or protein folding caused by the mutation cannot be completely ruled out.

Overall, the wild-type, C1 and C2 strains didn’t differ significantly in the phototactic activity among each other and in relation to WT (Fig. 4c), whereas strains with altered cation selectivity and/or kinetics showed 100-fold lower photosensitivity or even less and a complete lack of phototaxis in the case of the anion-conducting C2-E90R.

### Analysis of the ion selectivity of ChR mutants upon their heterologous expression

The reduction of photosensitivity observed in the point mutants prompted us to investigate a possible change in ion selectivity, specifically the conduction of Ca^2+^ as a cause for this phenomenon. Therefore, we performed electrophysiological measurements in ND7/23 cells expressing the respective mutations. Considering the low expression of ChR1 in mammalian cells^5,18^, we chose a chimera of ChR1 and ChR2 with the transmembrane helices 1–5 taken from ChR1 and helix 6 and 7 from ChR2 (*C1C2*−52^17^) to analyse the effect of the E162T mutation. Although C1C2 is not an exact match to ChR1, the pore-forming helices 1, 2 and 3, containing E162 and the active site residues are conserved and may give insight on the effects of the mutation on ion selectivity^20^.

**Fig. 5:**
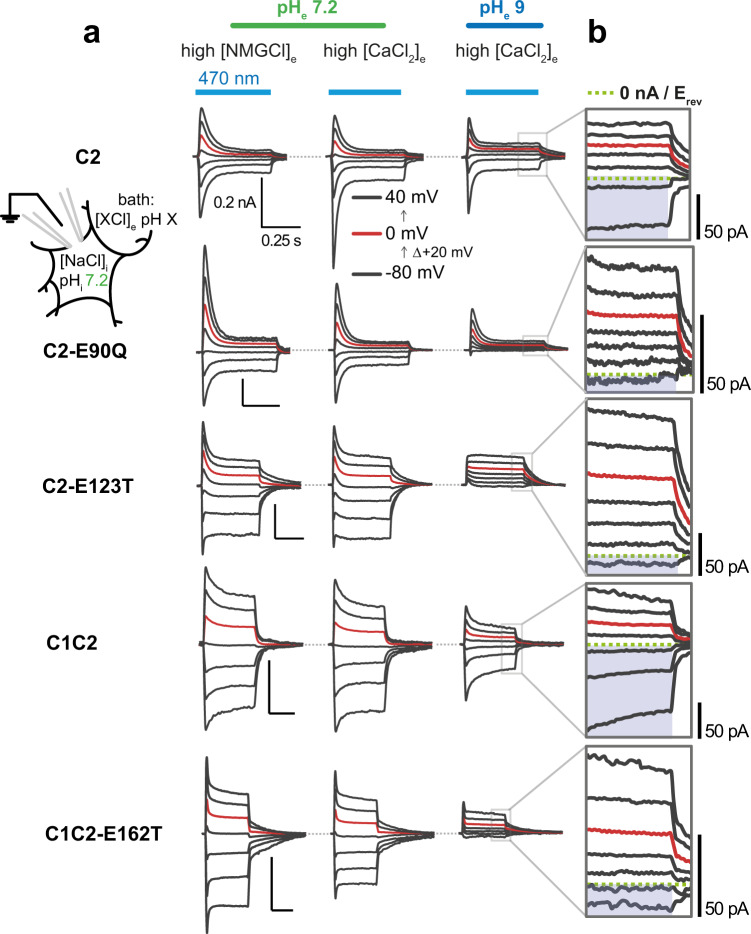
Electrophysiological characterization of the calcium permeation in C2, C1C2 and derived mutants. **a** Representative photocurrent traces of the denoted variant, recorded from −80 to +40 mV in 20 mV steps under different extracellular buffer conditions in ND7/23 cells. Red traces depict the current at 0 mV holding voltage. **b** Zoom in on the currents in high [CaCl_2_]_e_ at pH_e_ 9. The green dotted line indicates net-zero photocurrent. Inward calcium-driven currents are highlighted in violet.

We compared proton permeation using NMGCl_e_ in the external buffer with calcium permeation using CaCl_2e_ at a physiological pH_e_ of 7.2. Then, to observe calcium permeation without substantial proton contribution, the measurements were made at pH_e_ 9. In our electrophysiological analysis, we observed both inward and outward currents for C2 and C1C2 in the presence of extracellular Ca^2+^ at physiological pH (pH_e_ 7.2, Fig. 5a and Supplement Figs. 3, 4). This permeation was slightly inward rectified, suggesting a voltage-dependent cation transport in C2 and C1C2 (Supplement Fig. 4). Upon proton reduction at pH_e_ 9, the total current was lower but still observable (pH_e_ 9, Fig. 5a, b and Supplement Figs. 3, 4). It is important to note that C2 displayed a slightly better calcium conductance compared to C1C2, as evidenced by the increase in current size when adding extracellular calcium (compare NMGCl_e_ and CaCl_2e_ at pH_e_ 7.2, Fig. 5a, Supplement Figs. 3, 4) supporting earlier studies in *Xenopus* oocytes^17^. In contrast to C2, both the C2-E90Q and C2-E123T variants showed only slight differences in current size upon Ca^2+^ adding to the extracellular buffer (compare NMGCl_e_ and CaCl_2e_ at pH_e_ 7.2, Fig. 5a). Moreover, they exhibited negligible inward currents and a negative shift in reversal potential, *E*_rev_, at pH_e_ 9, indicating a strong reduction in calcium permeation for both mutants (high CaCl_2e_ pH_e_ 9, Fig. 5a, b and Supplement Figs. 3, 4). This is in line with a previous screening aiming at the reduction of calcium permeation, where specifically E90Q and E123 mutants were linked to reduced calcium conductance^32^. Like the C2 mutants, C1C2-E162T also exhibited both a negative shift in *E*_rev_ and minimal inward currents at high extracellular Ca^2+^ at pH_e_ 9, suggesting again a strongly decreased calcium permeation in comparison to the parental C1C2 (Fig. 5a, b and Supplement Figs. 3, 4).

All in all, the results of the electrophysiological study in the neuroblastoma ND7/23 cells reveal that the reduced phototactic sensitivity in the Chlamydomonas mutants C1-E162T, C2-E123T and C2-E90Q correlates with reduced Ca^2+^ permeation. The Na^+^ conductance, which is higher than Ca^2+^ and H^+^ conductance at neutral pH and relevant for optogenetic applications, is irrelevant for these studies and has not been measured, because Chlamydomonas lives naturally at low Na^+^ concentrations.

### Evaluation of photophobic response

**Fig. 6:**
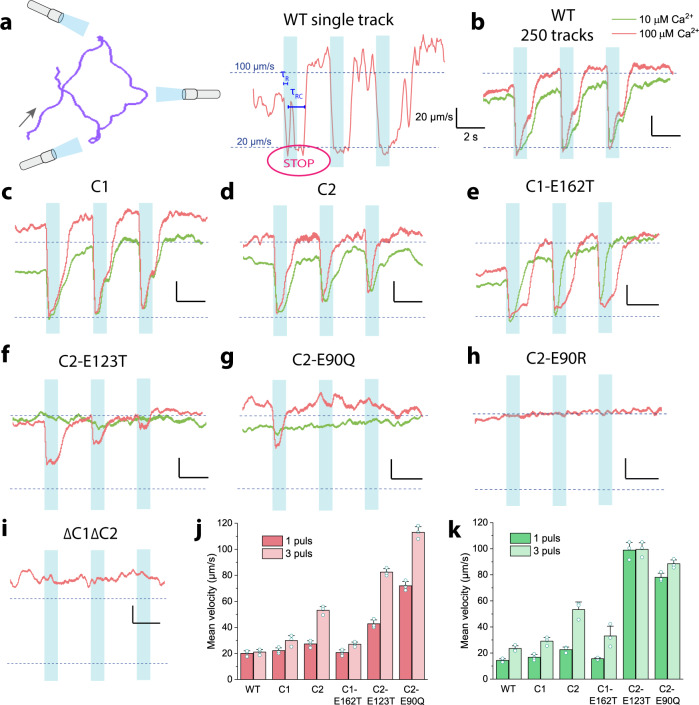
Photophobic response in *Chlamydomonas reinhardtii* cells to blue light pulses. **a** Representative track of a single wild-type cell illuminated with 470-nm light (1.75 × 10^20^ photons/m^2^s) three times for 1 s with 2 s dark periods at 100 µM Ca^2+^. Change of cell velocity upon illumination corresponding to a photophobic response (STOP) where τ_R_ is the reaction time and τ_RC_ is the recovery time. Velocity plots of 250 averaged tracks were obtained for the wild-type (**b**), C1 (**c**) and C2 (**d**) strains, point mutants C1-E162T (**e**), C2-E123T (**f**), C2-E90Q (**g**), C2-E90R (**h**) and the double knock-out ΔC1ΔC2 (**i**) at different Ca^2+^ concentrations. **j** Mean photophobic reaction velocity after the 1^st^ and the 3^rd^ light pulse at 100 µm Ca^2+^. **k** Mean photophobic reaction velocity after the 1^st^ and the 3^rd^ light pulse at 10 µm Ca^2+^. Lines and bars represent the mean value ± S.E. *N* = 3 experiments. Source data are available as a Source Data file.

To elaborate on the channelrhodopsins ion selectivity that affects the photophobic reaction and light adaptation of *Chlamydomonas reinhardtii*, we applied a single cell tracking technique. As seen from the recorded Chlamydomonas swimming patterns, dark-adapted wild-type cells consistently embraced an abrupt transient reverse swimming in response to 1 s light pulses with 2 s dark intervals (Fig. 6a). This so-called phobic or stop response was marked by an immediate reduction in velocity to 15 µm/s during backward swimming approximately 40 ms after the illumination start and a duration of about a half to one second, which depended mainly on such conditions as light intensity, wavelength and Ca^2+^ concentration^1^. The two distinctive inversion points can be precisely determined for an individual cell, whereas the recovery point is blurred for the population (Fig. 6a, b). In our setup, the first inversion point emerged 260 ms after the flash indicating a maximal probability for a cell to switch from forward to backward swimming and is defined as the reaction time (τ_R_), while the second inversion point appeared with an average 1.1 s delay. Finally, the recovery time (τ_RC_) of 1.16 s was derived from a difference between the first inversion point and the half-maximal recovery of the final swimming speed. After three consecutive flashes, the cell retained the same level of photophobic swimming speed of 15 µm/s.

In the cell population with variations in photosensitivity and velocity levels, the above-defined time parameters diverged. Here, the mean cell velocity following illumination was defined for 250 averaged tracks of cells that perform backward swimming and those that still might be swimming forward based on sensitivity differences plus variation of the eyespot orientation in respect to the incident light. After the first light pulse at standard (100 µM) and low (10 µM) Ca^2+^ all the strains (Fig. 6b–g) except C2-E90R (Fig. 6h) and ΔC1ΔC2 (Fig. 6i) exhibited a drop in the mean velocity indicating a photophobic response, which was less pronounced in C2-E123T (Fig. 6f) and C2-E90Q (Fig. 6g).

When comparing the mean velocities following the first and the third light pulses, wild-type cells showed hardly any reduction of photophobic response and overall low adaptation to changing light conditions at 100 µM Ca^2+^ (Fig. 6j). However, at lower Ca^2+^, the adaptation levels became more pronounced, and the mean velocity increased by 10 µm/s upon subsequent flashes (Fig. 6k). In the C1 and C2 strains at 100 µM Ca^2+^, the change was more pronounced with an increase of 8 µm/s for C1 and 25 µm/s for C2, consistent with the observed stronger reduction of current amplitudes in C2 and higher photocurrent desensitization in ND7/23 cells during illumination (Fig. 5a, b).

Again, adaptation levels slightly increased at lower extracellular Ca^2+^ when the average velocity changed by 13 µm/s for the C1 strain and 30 µm/s for the C2 strain. These observations prove the importance of Ca^2+^ as a charge carrier of intraflagellar currents and its involvement in the control of flagellar beating^33–35^. Furthermore, in the C1-E162T mutant, the adaptation levels at 100 µM Ca^2+^ were similar to the background strain C1 but slightly increased by 17 µm/s at low Ca^2+^. The ChETA mutant C2-E123T exhibited a drastic acceleration of the mean velocity following illumination both at standard and low extracellular Ca^2+^. Thus, the velocity increased by 41 µm/s, and at the low Ca^2+^, no stop response was recorded. These findings correlate with the fast channel opening and closing kinetics and, as a result, diminished Ca^2+^ conductance of the C2-ChETA mutant (Supplement Fig. 5). More intriguingly, the C2-E90Q mutant was less responsive and fully adapted already upon the second light pulse under standard and low Ca^2+^ conditions, hence, demonstrating the importance of H^+^ conductance for photo-orientation of *Chlamydomonas reinhardtii* that was previously only hypothesized from electrical measurements on the Chlamydomonas cell strain CW2 expressing only ChR1^8^. Thus, due to lack of E90 deprotonation and reduction of late photocurrent in the *syn*-photocycle, the cells adapted faster with no response to the second light pulse^22^. Finally, the Cl^−^-conducting C2-E90R strain completely lacked a photoresponse similar to the double knockout ΔC1ΔC2 (Fig. 6h, i). The reason for this is that the Nernst potential for chloride should be near the resting potential of the alga in freshwater lakes, as well as in our test medium (with a voltage around −120 mV according to Malhotra and Glass^36^), and therefore only small if any outward chloride currents are expected.

### Evaluation of electrical response

To monitor the light-induced electrical response of modified channelrhodopsins in vivo, we adapted a suction pipette technique that was applied first to photoreceptor cells in toad *Bufo marinus*^37,38^, to the alga *Haematococcus pluvialis*^39^ and later to cell-wall deficient *Chlamydomonas reinhardtii* strains^14^. To use the technique on wild-type cells and derived mutants, we employed gametolysin, an enzyme produced during mating of Chlamydomonas, to dissolve the cell wall and gain access to the cell plasma membrane^40,41^.

**Fig. 7:**
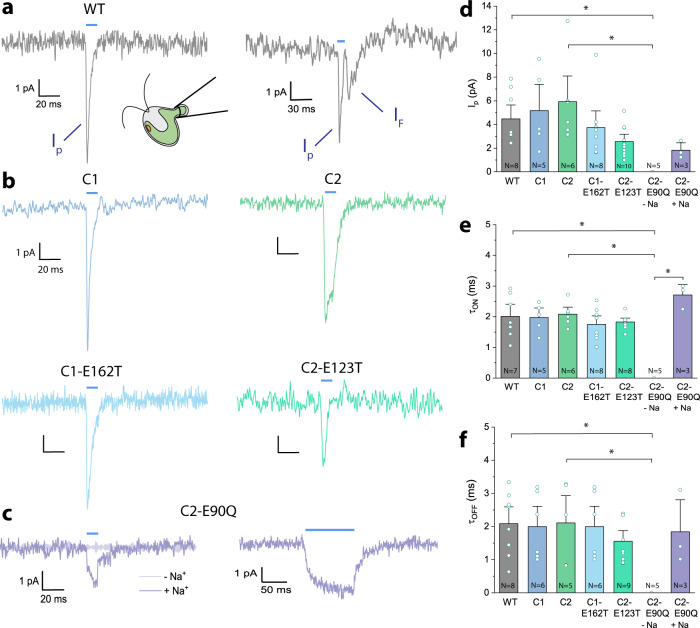
Electrical responses of *Chlamydomonas reinhardtii* cells to light pulses. **a** Representative traces of photoreceptor current (*I*_p_) and flagellar current (*I*_F_) in the wild-type. The cells were illuminated with 470-nm light for 10 ms (blue bar), and during measurements, a holding potential of −20 mV was applied. **b** Representative photocurrent traces of C1 and C2 strains, the double knock-out ΔC1ΔC2 and mutants C1-E162T, C2-E123T and C2-E90Q. **c** Representative photocurrent traces of C2-E90Q mutant at the standard Ca^2+^ concentration and at the addition of Na^+^ upon illumination for 10 and 100 ms. **d** Peak photocurrent amplitudes measured at the standard Ca^2+^ concentration and at the addition of Na^+^ for C2-E90Q upon illumination for 10 ms. **e** Kinetics of the peak photocurrent rise. **f** Kinetics of the photocurrent decay. Photocurrent kinetics were fitted mono-exponentially. Lines and bars represent the mean value ± S.E. Significant differences between the mean values are marked with * (*p* = 0.003 in **d**, **e** and *p* = 0.006 in **f**, one-way ANOVA with posthoc Tukey test). *N* corresponds to a number of biological replicas. Source data are available as a Source Data file.

A membrane region of about 30 % of the whole coverage was sucked into the pipette, and the current was measured during stimulation with a 10 ms light pulse. During these measurements, the membrane potential (*E*_m_) of the cell was not held constant. Therefore, the recorded photocurrents were directly affected by changes in *E*_m_. Furthermore, the composition of the intracellular ions was not controlled. Due to the small algal size, photoinduced currents were detected from any location on the surface as capacitive currents^8^. Moreover, depending on the position of the eyespot or flagella inside the pipette, the signals may have an opposite sign^7^. However, since the photocurrent kinetics didn’t differ at different measuring positions, the signals of an opposite sign indicate the same electrical response. In general, one or two photocurrent components were detected in a cell in response to photo-stimulation. The first component arises at the onset of illumination and is believed to localize in the eyespot. Therefore, it has been assigned as the photoreceptor currents (*I*_p_)^7,14,33^ (Fig. 7a). The second component arises with a delay after *I*_p_ decayed. Previously, it was identified as flagellar currents (*I*_F_)^14^. These last for a few milliseconds, exhibiting slow decay kinetics. Earlier studies suggested that these flagellar currents were Ca^2+^-carried and originated from voltage-gated flagellar channels^7,14,33^.

Due to the low seal resistance between cell and pipette, the measured photocurrent amplitudes were much smaller than the real current, reaching maximally 12 pA. Transient *I*_p_ amplitudes of the wild-type, C1 and C2 strains averaged at 4.5 – 6 pA but were slightly reduced for C1-E162T (4 pA) and C2-E123T (2.5 pA) (Fig. 7a, b, d). This is in agreement with the electrophysiological studies in the ND7/23 cells (Supplement Figs. 3–5). More intriguingly, no photocurrents were measured for the E90Q cells at the standard Ca^2+^ conditions but were restored after the addition of Na^+^ (Fig. 7c, d). Considering reduced H^+^ conductance in the E90Q mutant, the substitution of protons with other monovalent ions, Na^+^ here, and observed recovered photocurrents confirm the important role of the H^+^ conductance in light-induced behavioural responses of Chlamydomonas wildtype cells (Figs. 4, 6). Further, the opening kinetics of *I*_p_ (τ_ON_) didn’t differ significantly in the wild-type, C1 and C2 strains with the respective peak values monitored at around 2 ms (Fig. 7e). Surprisingly, τ_ON_ in the C2-E90Q strain in presence of Na^+^ decelerated to 2.7 ms. Furthermore, except for C2-E90Q, *I*_p_ inactivated after reaching its maximum amplitude. This inactivation may be attributed to other cellular processes triggered by *I*_p_. While *I*_p_ inactivated with a kinetic (τ_OFF_) of approximately 2 ms in the wild-type as well as the C1, C2 and C1-E162T strains, the inactivation shifted to 1.8 ms in the C2-E90Q strain and 1.6 ms in the C2-E123T strain (Fig. 7f).

## Discussion

The green unicellular algae *Chlamydomonas reinhardtii* is a unique model organism, for which a set of tools for genetic manipulation is now available. With its functional eye, a locomotion system and active photosynthetic complexes, it stands at the intersection of the animal and plant world. Light-gated ion-conducting channelrhodopsins ChR1 and ChR2 are invaluable for this algae’s ability to perceive and process light signals for orientation. Around 900 ChRs have been identified in a large number of algal genomes and some algal viruses so far, with quite a variable selectivity ranging from highly H^+^ selective to highly Cl^−-^ or K^+^ -selective with different degrees of inactivation and the physiological meaning of the selectivity variation is unclear^42–44^. ChRs have been poorly analyzed in vivo, mainly due to the difficulties of algal cultivation and genetic modifications of selected genes in *C. reinhardtii* and all relatives.

In this study, we presented a technique for precise gene targeting and editing that has been employed for the integration of single point mutations at various locations in the Chlamydomonas genome. The technique is based on the utilization of the CRISPR-Cas9 editing tool for the initiation of double-stranded breaks in genomic DNA, their subsequent repair via homologous recombination with a donor DNA, including a single nucleotide exchange, and rapid identification of the mutants by colony PCR. We proceeded with genomic editing in two ways and successfully obtained strains with point mutations in channelrhodopsins. However, in the end, the one-step approach proved to be less time-consuming and, therefore, more efficient.

In the wild-type strain СС125, ChR1 is highly expressed, but ChR2 abundance is very low in vegetative cells and gametes (Fig. 3, Supplement Fig. 6). In other tested Chlamydomonas strains, the ratio of both proteins slightly varies depending on the growing conditions that promote asexual or sexual reproduction^2,18,24,45^. Also, in the multicellular algae *Volvox carteri*, both channelrhodopsins are differentially expressed during day and night but only in biflagellate somatic cells and are not present in asexual reproductive cells. However, after the addition of a sex inducer protein, the expression of ChRs, primarily ChR2, increased in reproductive cells as well^46^. Interestingly, out of the strains tested so far only in the Chlamydomonas wild-type CC125, ChR2 is strongly upregulated in the absence of ChR1 (Fig. 3), which allowed us to study the function of ChR2 in vivo. Neither in the previously generated antisense ChR1 transformants in the CW2 strain^18^ nor in ChR1 knock-down in the 495 strain where ChR1 expression was suppressed by an inverted-repeat genetic construct^2,45^, nor in the CRISPR-Cas9-generated ChR1 knock-out in CC3403^24^ such a drastic upregulation of ChR2 has been observed.

The physiological responses of the mutant strains with altered photocycle kinetics and channel ion selectivity were studied through a set of biophysical methods, including light scattering (Fig. 4), single-cell tracking (Fig. 6) and cell-attached photocurrent recording (Fig. 7). Mutation of a retinal Schiff base counterion in the C1-E162T and C2-E123T strains resulted in a reduced Ca^2+^ selectivity (Fig. 5) which further led to a reduction of the photoinduced modulation in flagella beating patterns and phototaxis, especially at low light intensity (Figs. 4, 6). Both a reduced Ca^2+^, as well as H^+^ conductance^21,22^ were found for C2-E90Q (Fig. 5 and Supplement Figs. 3, 4) resulting in a behavior similar to C1-E162T and C2-E123T (Figs. 4, 6, 7). The C2-E90R strain, on the other hand, was not photosensitive at all, lacking both phototaxis and photophobic responses, demonstrating that the almost completely impaired cation conductance^23^ cannot be compensated for by passive chloride efflux. It is already known that Chlamydomonas naturally contain a voltage-gated chloride channel in the periphery of the eyespot^47^ but a chloride efflux is only detected at nearly zero chloride in the extracellular medium (E. Govorunova and P. Hegemann, unpublished).

Moreover, due to the reduced Ca^2+^ selectivity and shorter open state life times both ChETA mutants showed smaller photocurrents (especially at low Ca^2+^), faster recovery from a photophobic reaction and faster adaptation to repetitive light stimuli when compared to the respective background C1 and C2 strains. The effect is more pronounced in C2-E123T due to the smaller total photoreceptor current compared to the wild-type and the lack of photocycle extension upon depolarization^48^. The complete absence of phobic responses for E90Q cells is again explained by the almost complete absence of the conductance for both H^+^ and Ca^2+^ in the test buffer caused by impairment of E90 deprotonation^21,22^. In wild-type ChR2, the rearrangement of hydrogen bonds between E90 and E123 enables water influx and deprotonation of E90^22^. The E123T mutation also prevents deprotonation of E90 and contributes to the suppression of late photocurrents^22^. In addition, all the mutants and respective background strains accelerated the adaptation rates at low extracellular Ca^2+^, highlighting the importance of Ca^2+^-driven currents for the alteration of the flagella beating. Moreover, the mere presence of Ca^2+^ conductance is sufficient for the phototactic activity, whereas for the photophobic reaction the Ca^2+^ and H^+^-driven photocurrents need to be high enough to trigger the opening of voltage-gated Ca^2+^ channels in the flagella^12,14,15,33^ (Supplement Fig. 7). Thus, the flagellar photocurrents are a response to high-intensity light and high Ca^2+^ concentration and reflect the processes underlying a photophobic reaction. However, not every current recording showed the flagellar current component. This might be connected to an incomplete cell wall removal during the gametolysin treatment. All in all, slightly faster opening and closing kinetics of the channel in the ChETA mutant strains together with reduced photocurrent amplitudes fit well with electrophysiological studies of the respective mutants in host cell systems and contribute to the low phototactic sensitivity, reduced photophobic reaction and faster light adaptation. Detection of photocurrents and phobic reactions of E90Q at only high Na^+^ supports the hypothesis that H^+^ and Ca^2+^ are crucial for naturally occurring photo behavioral responses of *Chlamydomonas reinhardtii*.

With our studies, we not only clarified the different physiological functions of ChR1 and ChR2, we also demonstrated the potency of in vivo site-directed point mutation. The presented method can be used for very specific and targeted modifications on a gene level to unravel the function of various proteins and their contribution to physiological responses of *Chlamydomonas reinhardtii* and can be extended to other algal or flagellated organisms.

## Methods

### Chlamydomonas strains and culture preparation

Motile *Chlamydomonas reinhardtii* wild-type strain CC125 was obtained from the Chlamydomonas Resource Center (http://www.chlamycollection.org). Cells were grown in standard Tris-acetate phosphate (TAP) medium^49^ under continuous cool fluorescent white light of 40–60 µE·m^−2^s^−1^ at 110 rpm at 22 °C. Alternatively, the cultures were synchronized for the transformation in cycles of 14 h at 25 °C in light and 10 h at 18 °C in darkness.

For protein analysis, phototactic essay, cell tracking and detection of electrical responses the cell cultures of the wild-type and mutant strains were transferred to NMM medium (80 μM MgSO_4_, 100 μM CaCl_2_, 3.1 mM K_2_HPO_4_, and 3.4 mM KH_2_PO_4_, pH 6.8) and incubated for 24 hours at standard light conditions to induce gametogenesis and to ensure equal protein expression levels in a cell population. For the behavioral studies, the algae suspension was kept in darkness for 1 hour before the measurements.

### Mammalian cell culture (ND7/23)

ND7/23 cells (Sigma-Aldrich, St. Louis, MO, USA) were grown on glass coverslips coated in poly-D-lysine, which were placed in 35 mm Petri dishes containing Dulbecco’s Modified Eagle Medium (DMEM; Biochrom GmbH) with 5% (v/v) fetal bovine serum (FBS superior; Biochrom, Berlin Germany), glutamine (Biochrom, Berlin Germany) and 100 µg/ml penicillin/streptomycin (Biochrom, Berlin Germany). Moreover, the growth media contained 1 µM all-*trans* Retinal. To transiently transfect cells, 6 µl FuGENE HD transfection reagent (Promega, Madison, WI) was incubated with 2 µg of vector DNA in 250 µl DMEM for 15 minutes and added to the cells two days prior to measurements.

### Cas9 protein purification

Recombinant *Streptococcus pyogenes* Cas9 protein was expressed from the plasmid *pET-28b-Cas9-His* (https://www.addgene.org/47327/) in *Escherichia Coli* strain Rosetta2(DE3)pLys2 at 20 °C and then purified by a Ni-NTA column following the published protocol^50^.

### Transformation of *Chlamydomonas reinhardtii* cells by CRISPR-Cas9 technique

Mutants with disrupted ChR genes, as well as mutants with single amino acid changes, were obtained by using the CRISPR-Cas9 gene-editing technique as described previously^24^. The Cas9 protein together with a guide RNA (gRNA) forms a ribonucleoprotein (RNP) complex. The gRNAs were combined from a scaffold RNA (tracrRNA) with a constant sequence and a target sequence RNA (crRNA) specific for a chosen target site. The crRNAs were designed for each target site using search tools *CRISPR-P*^51^ and *CRISPOR*^52^. Two complementary homology-directed repair (HDR) donor oligonucleotides consisted of either a FLAG insert for gene knockouts or a single amino acid exchange for point mutations. The sequences of both single-stranded (ss) oligonucleotides were 90 bp and had phosphorothioate bonds (PTO) 3′ and 5′ end bases for protection. The tracrRNAs, crRNAs and ss-HDR donor oligonucleotides were ordered from Integrated DNA Technologies (IDT, Coralville, USA).

To obtain a gRNA, equal molar amounts of crRNA and tracrRNA were annealed in DUPLEX buffer (IDT, Coralville, USA) at 95 °C for 2 min followed by cooling at a rate of 0.1 °C/min. Next, the Cas9 protein was mixed with the gRNA in 1xBuffer O (Thermo Fischer Scientific, Walltham, USA) to a final concentration of 3 µM each and incubated at 37 °C for 15 min to obtain the RNP complex. The cell culture was grown in a synchronized light/dark cycle and transferred into MAX Efficiency Transformation Reagent for Algae (Thermo Fischer Scientific, Walltham, USA) supplemented with 40 mM sucrose with the final concentration 1×10^8^ cells/ml. Subsequently, the cell suspension was exposed to a heat shock at 40 °C for 30 min at 350 rpm. After 1 h recovery, the cells were mixed with 5 μl of 3 μM RNP, 20 pmol ss-HDR oligonucleotides and 0.3 μg of one of the selection marker plasmids pAphVII (pPH360), pAphVIII (pPH075) or Zeocin (pPH063) and then electroporated using the NEPA21 Super Electroporator (Nepa Gene Co., Chiba, Japan). After the transformation, the cells were selected on TAP plates containing 10 µg/ml of the respective selection marker.

### Screening of Chlamydomonas mutants

PCR amplification of genomic Chlamydomonas DNA was performed using Phire Plant Direct PCR Master Mix (Thermo Fischer Scientific, Walltham, USA). Picked colonies were transferred in 180 μl of TAP medium in 96-well plates. 40 μl of the grown green culture were centrifuged at 2000× *g* for 10 min at room temperature. After the supernatant was discarded the cell pellet was resuspended in 20 μl of dilution buffer, incubated for 5 min at room temperature and centrifuged at 4000× *g* for 10 min. 10-μl PCR Master Mix contained 1 μl of DNA extract solution reaction, 10 pmol oligonucleotides, 1 M betaine and 1×Phire Plant Master Mix. A PerfectBlue Maxi ExW electrophoresis system (Peqlab, VWR, USA) was used to analyze the PCR products with 1–3% Tris-borate-EDTA agarose gels.

### Preparation of plasmids for ND7/23 cells

Human codon-optimized sequences for ChR2 were cloned into pmCherry-N1, as previously described^53^. The sequence *C1C2*-52 with enhanced membrane targeting was cloned via Gibson assembly^54^ pCDNA3.1 in frame with TS-mScarlet-ER (TS and ER sequence are as described in Grimm et al.^55^). Site-directed mutagenesis of ChR2 and *C1C2*-52 was performed via Quikchange using a *Pfu* polymerase (Agilent Technologies, Santa Clara, CA).

### Protein immunoblotting

Total cell proteins were separated by sodium dodecyl sulfate-polyacrylamide gel electrophoresis using 4–15% gradient Mini PROTEAN TGX precast protein gels (Bio-Rad, Hercules, USA) and transferred onto low fluorescence polyvinylidene difluoride membranes using a Trans-Blot Turbo transfer system (Bio-Rad, Hercules, USA). Blots were incubated overnight with an affinity-purified anti-ChR1 (1:1000) rabbit monoclonal antibody (provided by Dr Suneel Kateriya) and commercial anti-ChR2 (1:200) mouse monoclonal antibody (PROGEN, Heidelberg, Germany). Secondary antibodies horseradish peroxidase-conjugated ECL anti-rabbit IgG (1:2000) and anti-mouse IgG (1:5000) (Invitrogen, Waltham, USA) were allowed to bind for 2 h. Clarity ECL Western substrate-induced luminescence was detected using the ChemieDoc MP system (Bio-Rad, Hercules, USA). Then the membranes were rinsed extensively with PBS-T (phosphate buffered saline with 0.1 % Tween-20; Sigma-Aldrich, St. Louis, USA) and incubated overnight with an anti-AtpB (beta subunit of ATP synthase) rabbit polyclonal primary antibody (1:2000) (Agrisera, Vännäs, Sweden) which was used as a loading control. The secondary antibody ECL anti-rabbit IgG was used in concentration 1:7500. The immunoblotting data were analysed with the software ImageLab 2017 (Bio-Rad, Hercules, USA).

### Phototaxis assay in *Chlamydomonas reinhardtii* via light scattering technique

The populational phototactic response was monitored with a custom-made light-scattering apparatus^18,31^. Before the measurements, the algae suspension was diluted to the concentration 1 × 10^6^ cells/ml and kept in darkness for 1 hour. The light from an infrared LED (840 nm) was scattered from the cell suspension in a fluorescence cuvette (20 mm, SOG 3; Starna, Pfungstadt, Germany) on infrared-sensitive photodiodes. The current produced by the IR diode that corresponds to the intensity of the scattered light was processed by a current-voltage amplifier (10^6 ^V/A) with a 10 Hz low pass filter using the signal conditioner CyberAmp 320 (Molecular Devices, Sunnyvale, USA). The data acquisition system Digidata 1322 A and the software pClamp9 (Molecular Devices, Sunnyvale, USA) were used to process the voltage proportional to the intensity of the scattered light. To trigger phototactic response, an LED of 470 nm (Quadica Developments, Lethbridge, Canada) was used, and light intensities were adjusted using neutral density filters (AHF Analysetechnik, Tübingen, Germany). Absolute irradiance values were determined using an optometer P9710 (Gigahertz Optik, Türkenfeld, Germany).

### Single cell tracking in Chlamydomonas reinhardtii

A cell suspension in NMM with the concentration of 1 × 10^5^ cells/ml was incubated for 1 hour in darkness. Then, the cells were imaged in a fluorescence cuvette (1 mm, G1; Starna, Pfungstadt, Germany) with four polished sides under an Olympus IX70 microscope (Olympus, Waltham, USA) with a dark-field condenser Olympus 4-4CDB-2 and a red 645 RG filter (AHF Analysetechnik, Tübingen, Germany). The phototactic and photophobic responses were triggered by 1 s flashes of a 470 nm LED (Quadica Developments, Lethbridge, Canada), which was fixed parallel to the cuvette and controlled by the data acquisition system Digidata 1550B and the software pClamp10 (Molecular Devices, Sunnyvale, USA). The movement patterns were recorded by a high-speed camera pco.panda 4.2 (PCO, Kelheim, Germany) and analysed with a plugin TrackMate v4.0.1 & v7.6.1^56^ of an opensource software ImageJ2 as well as Origin 2019 (OriginLab). Within the plugin, the filters for duration and displacement of tracks were applied. Light intensity was measured in the sample plane with an optometer P9710 (Gigahertz Optik, Türkenfeld, Germany).

### Whole-cell patch-clamp recordings in ND7/23 cells

Fire-polished, borosilicate patch pipettes (Science Products GmbH, Hofheim, Germany) were pulled with a P-1000 micropipette puller (Sutter Instrument, Novato, USA). Pulled pipettes had an access resistance of 1.5–3 MΩ. Transfected ND7/23 cells were measured in whole-cell configuration at 24 °C. Membrane resistance was generally > 0.5 GΩ, and access resistance was generally <10MΩ. A pE-4000 CoolLED (CoolLED, Andover, UK) was used for fluorescence and channelrhodopsin excitation. An Axiovert 100 TV inverted microscope (Carl Zeiss, Oberkochen, Germany) was used to search for fluorescent cells through a 40×/1.0 water objective (Carl Zeiss, Oberkochen, Germany). The CoolLED light path was coupled to the microscope.

An ELC-03XS amplifier (npi Electronic, Tamm, Germany) was used to amplify electrical recordings, and they were subsequently digitized using a Digidata 1440 A (Molecular Devices, Sunnyvale, CA). An AgCl electrode enveloped in agar and containing 140 mM NaCl was submerged in the bath solution to function as a reference electrode. For buffer exchange, a perfusion system (Ringer-Bath-Handler, Lorenz Meßgerätebau, Germany) was used. The 470 nm channel of the CoolLED system with an intensity of 2.9 mW/mm² was employed for ChR activation. Current-voltage measurements were performed using 500 ms LED flashes to induce currents, which were recorded from −80 to +40 mV in 20 mV steps. Automatization of recordings was accomplished via the Clampex software suite (Molecular Devices). Clampex was also used to precorrect liquid junction potentials in measurement protocols. Buffers used in experiments are listed in Supplement Table 1.

Data was analyzed using Clampfit 10.4 (Molecular Devices) and Origin 2017 (OriginLab, Northampton, MA). Stationary photocurrents were used for the determination of ion selectivity, which was calculated by averaging the photocurrents in the last 50 ms of illumination. I-V recordings were normalized to the stationary photocurrent at −80 mV holding potential at high NaCl_e_, pH 7.2. Reversal voltages were extrapolated by linear regression of the 2 data points closest to 0 pA. τ-values for kinetic calculations were obtained via a bi-exponential fit of the currents at −80 mV and high NaCl_e_, pH 7.2.

### Suction pipette recordings in *Chlamydomonas reinhardtii*

The cells grown in the NMM medium were first pre-selected for high photosensitivity and then treated with a gametolysin for 1 hour in darkness to dissolve the cell wall. The protocol for gametolysin preparation can be found elsewhere^57^. Before the measurement, the cells were resuspended in a measuring buffer including 5 mM Hepes, 10 mM HCl, 1 mM KCl, 0.3 mM CaCl_2_ and 0.2 mM Bapta at pH 6.8. For the Na^+^ content, 10 mM NaCl was used and the pH was adjusted to 6.8 with HCl. Then, the cells were fixed on a glass coverslip with a poly-D-lysine coating and imaged under an Axiovert 100 TV microscope (Carl Zeiss, Jena, Germany) with a red 645 RG filter (AHF Analysetechnik, Tübingen, Germany). The illumination was done for 10 ms with 470-nm light using pE-400 CoolLED System (CoolLED, Andover, UK) at the intensity 22.66 × 10^26^ photons/m^2^s (illuminated area 0.0346 mm^2^). Light intensity was measured in the sample plane with an optometer P9710 (Gigahertz Optik, Türkenfeld, Germany). The borosilicate-based patch pipettes with a resistance of 30–50 MΩ were fire-polished and filled with a measuring buffer. A 140 mM NaCl agar bridge was used as the reference electrode. The membrane resistance was generally 80–150 MΩ. The recorded signals were amplified and filtered with a 10-kHz low-pass Bessel filter using an ELC-03XS (npi Electronic, Tamm, Germany), digitized with DigiData 1400 A (Molecular Devices, Sunnyvale, USA) and acquired using Clampex 10.4 (Molecular Devices, Sunnyvale, CA). During measurements, a holding potential of −20 mV was applied. Six sweeps for each biological replica were smoothed and averaged. Photocurrent inactivation and growth were fitted mono-exponentially. The Software Clampfit 10.4 (Molecular Devices, Sunnyvale, CA) and Origin 2019 (OriginLab) were applied for analysis of electrophysiological recordings.

### Reporting summary

Further information on research design is available in the Nature Portfolio Reporting Summary linked to this article.

## Supplementary information

Supplementary Information

Reporting Summary

## Data Availability

Data supporting the findings of this manuscript are available from the corresponding authors upon reasonable request. Source data underlying Figs. 3, 4, 6, 7; and Supplementary Figs. 3–6 are available as a Source Data file. Source data are provided with this paper.

## Source data

Source Data
