# Supplementary material for: Gating and ion selectivity of Channelrhodopsins are critical for photo-activated orientation of Chlamydomonas as shown by in vivo point mutation
Source: Nat Commun. 2022 Nov 25;13:7253. doi: 10.1038/s41467-022-35018-6 (PMC9700795; doi:10.1038/s41467-022-35018-6)
Supplement: Supplementary file 1 — Supplementary Information [file 41467_2022_35018_MOESM1_ESM.pdf]

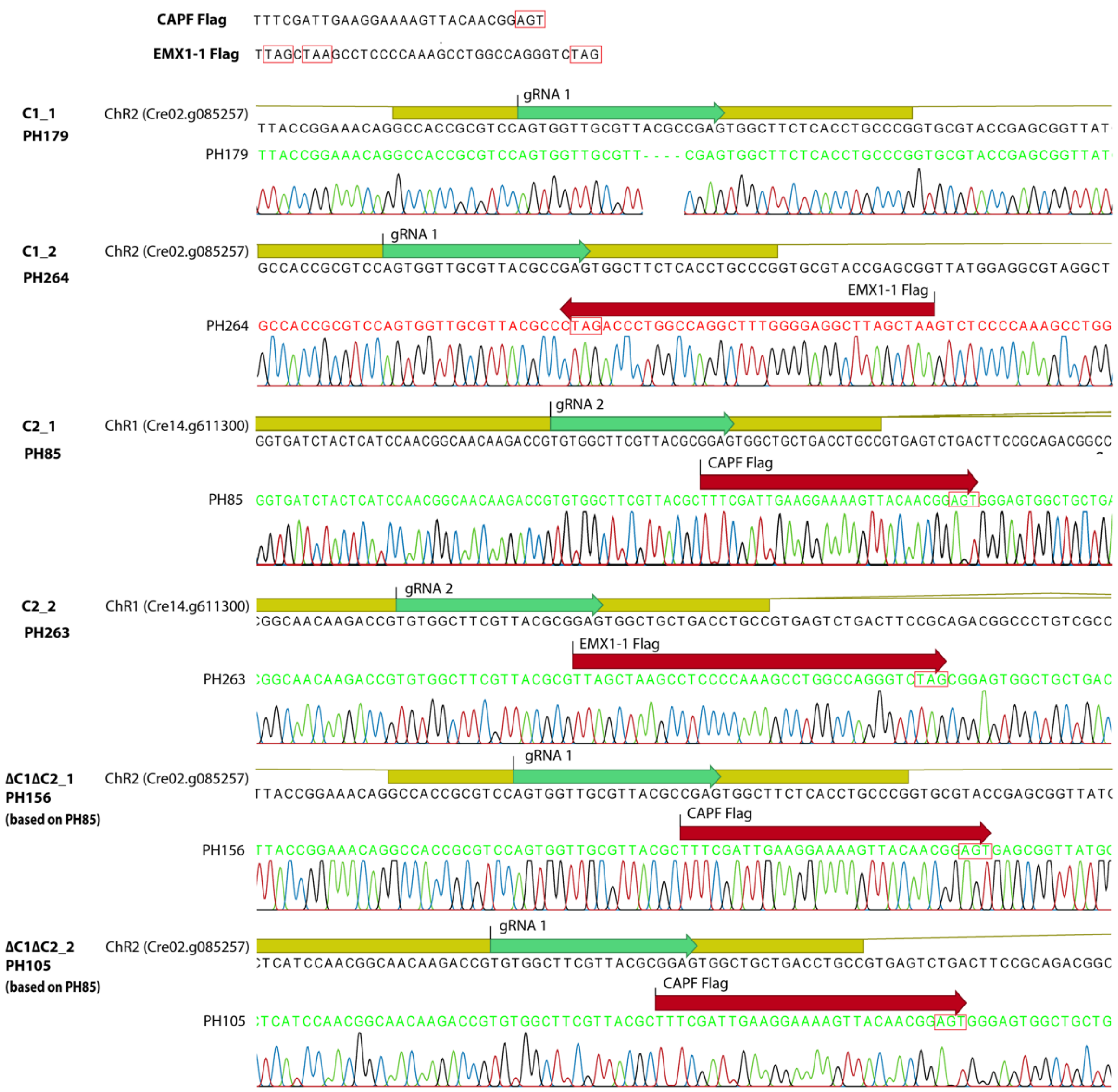

Supplementary Figure 1. Next Generation Sequencing of Chlamydomonas C1, C2 strains and the double knock-out ΔC1ΔC2. To generate a gene knock-out, a unique synthetic 29-31 bp DNA sequence called CAPF and EMX1-1 FLAG was integrated in the target site. The FLAG sequence includes stop codons (red box), leading to termination of translation. The single ChR knock-out strains PH85, PH264, PH263 were obtained by a clear insertion of a FLAG sequence in the target site, while the knock-out strain PH179 had a frame shift in the ChR2 gene. The double knock-outs ΔC1ΔC2 strains were based on the strain PH85 where subsequently ChR2 was inactivated with an introduction of the CAPF FLAG sequence.

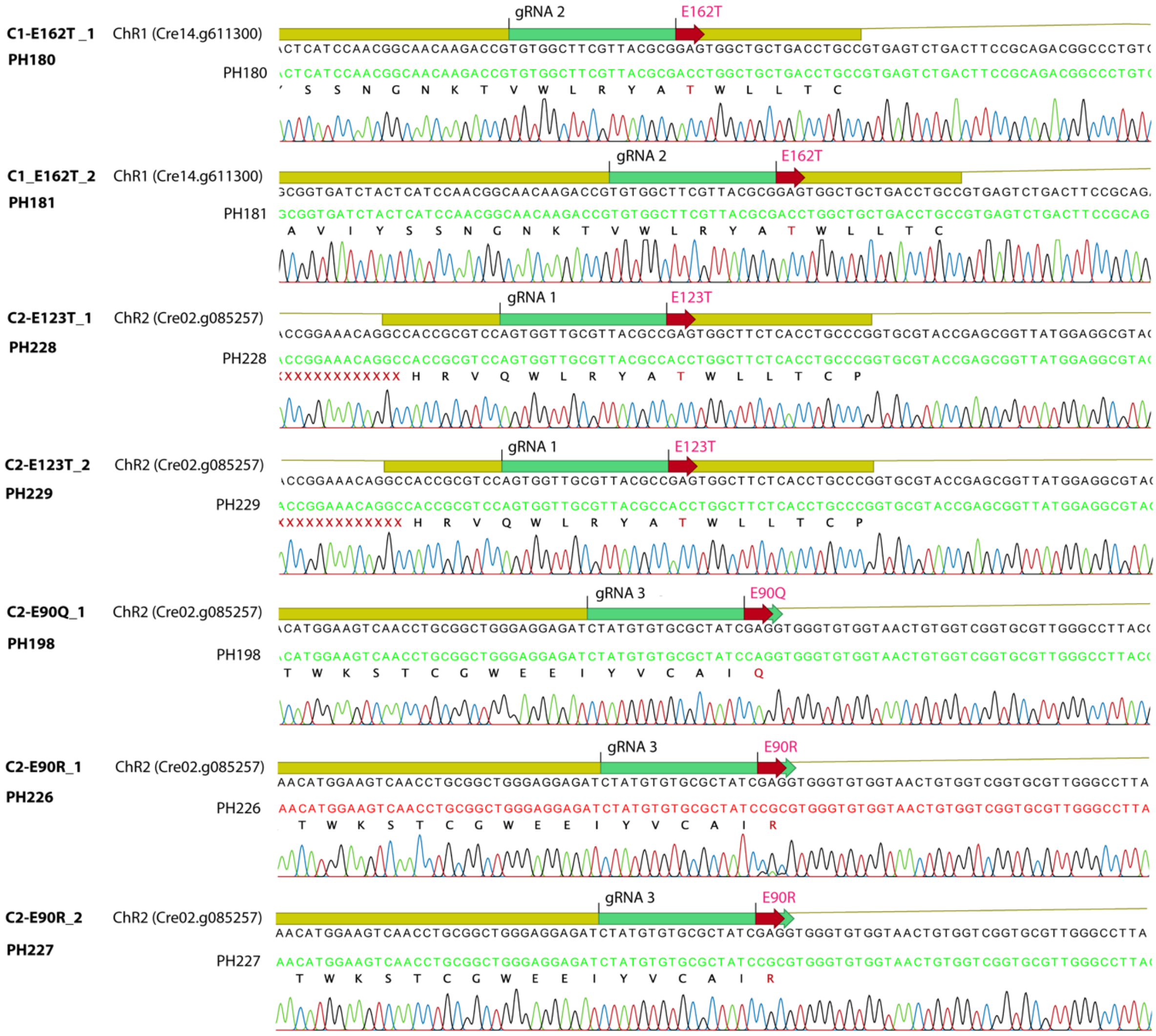

Supplementary Figure 2. Next Generation Sequencing of the Chlamydomonas point mutant strains C1-E162T, C2-E123T, C2-E90Q and C2-E90R.

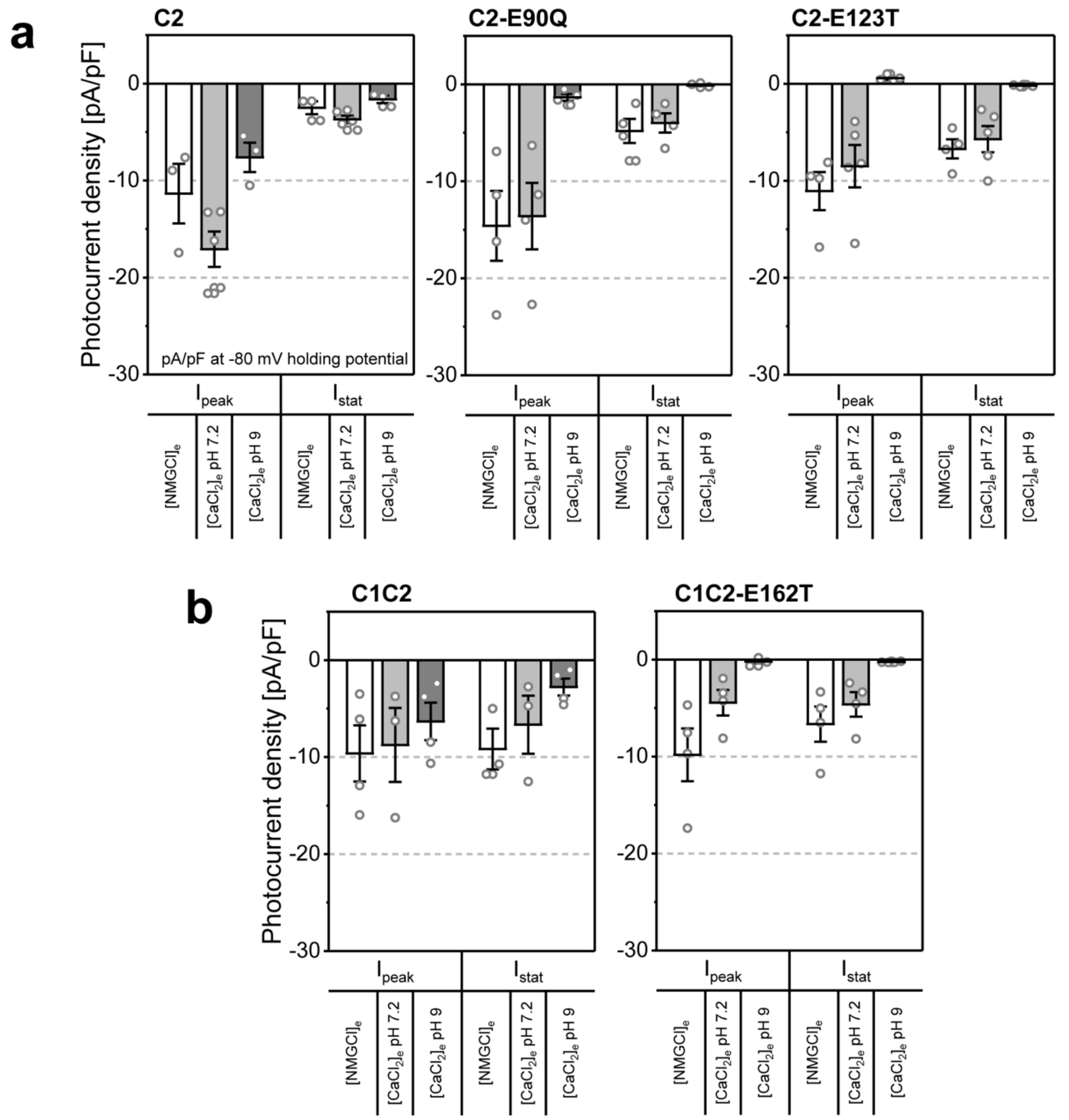

Supplementary Figure 3. **a** Peak and stationary photocurrent densities of C2 and derived mutants under the denoted buffer conditions (at -80 mV holding potential). **b** Peak and stationary photocurrent densities of C1C2 and derived mutants under the denoted buffer conditions (at -80 mV holding potential). Lines and bars represent the mean values ± S.E.M and dots depict single values. N is a number of biological replicas. For C2: N = 3 at [NMGCl]$_e$ & at [CaCl$_2$]$_e$ pH 9 and N = 5 at [CaCl$_2$]$_e$ pH 7.2; C2-E90Q: N = 4 under all 3 ionic conditions; C2-123T: N = 4 at [NMGCl]$_e$ & at [CaCl$_2$]$_e$ pH 9 and N = 5 at [CaCl$_2$]$_e$ pH 7.2; C1C2: N = 4 at [NMGCl]$_e$ & at [CaCl$_2$]$_e$ pH 9 and N = 3 at [CaCl$_2$]$_e$ pH 7.2; C1C2-E162T: N = 4 under all 3 ionic conditions.

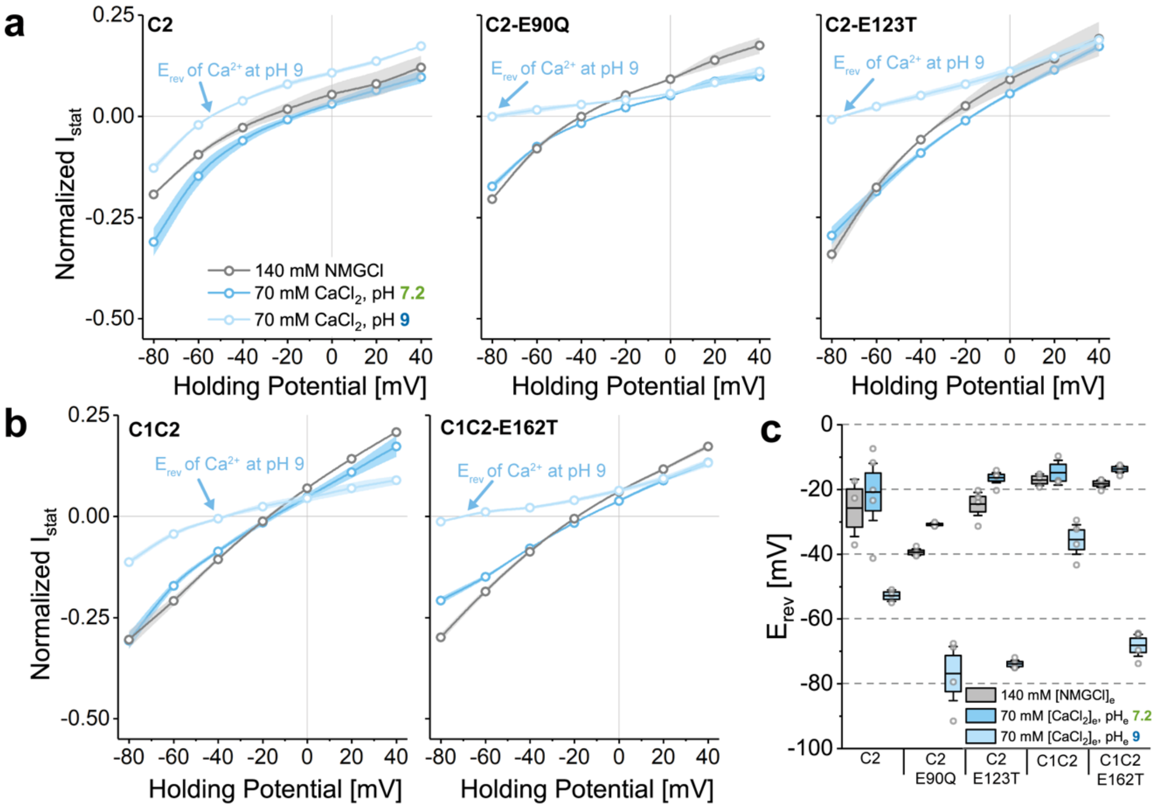

Supplementary Figure 4. **a** I-V relationships of C2 and derived mutants at the designated ionic conditions. Dots represent mean values, and the shadows represent the S.E.M. b I-V relation ships of C1C2 and derived mutants at the designated ionic conditions. Dots represent mean values and shadows the S.E.M. c Estimated reversal potentials for constructs measured in ND7/23 cells. Boxes: Middle line represent mean values, upper and lower box ranges denote S.E.M and whiskers represent S.E.M. x 1.5. N is a number of biological replicas. For C2: N = 3 at [NMGCl]e & at [CaCl2]e pH 9 and N = 5 at [CaCl2]e pH 7.2; C2-E90Q: N = 5 under a ll 3 ionic conditions; C2-123T: N = 4 at [NMGCl]e & at [CaCl2]e pH 9 and N = 5 at [CaCl2]e p H 7.2; C1C2: N = 3 at [NMGCl]e & at [CaCl2]e pH 7.2 and N = 4 at [CaCl2]e pH 9; C1C2-E16 2T: N = 4 under all 3 ionic conditions.

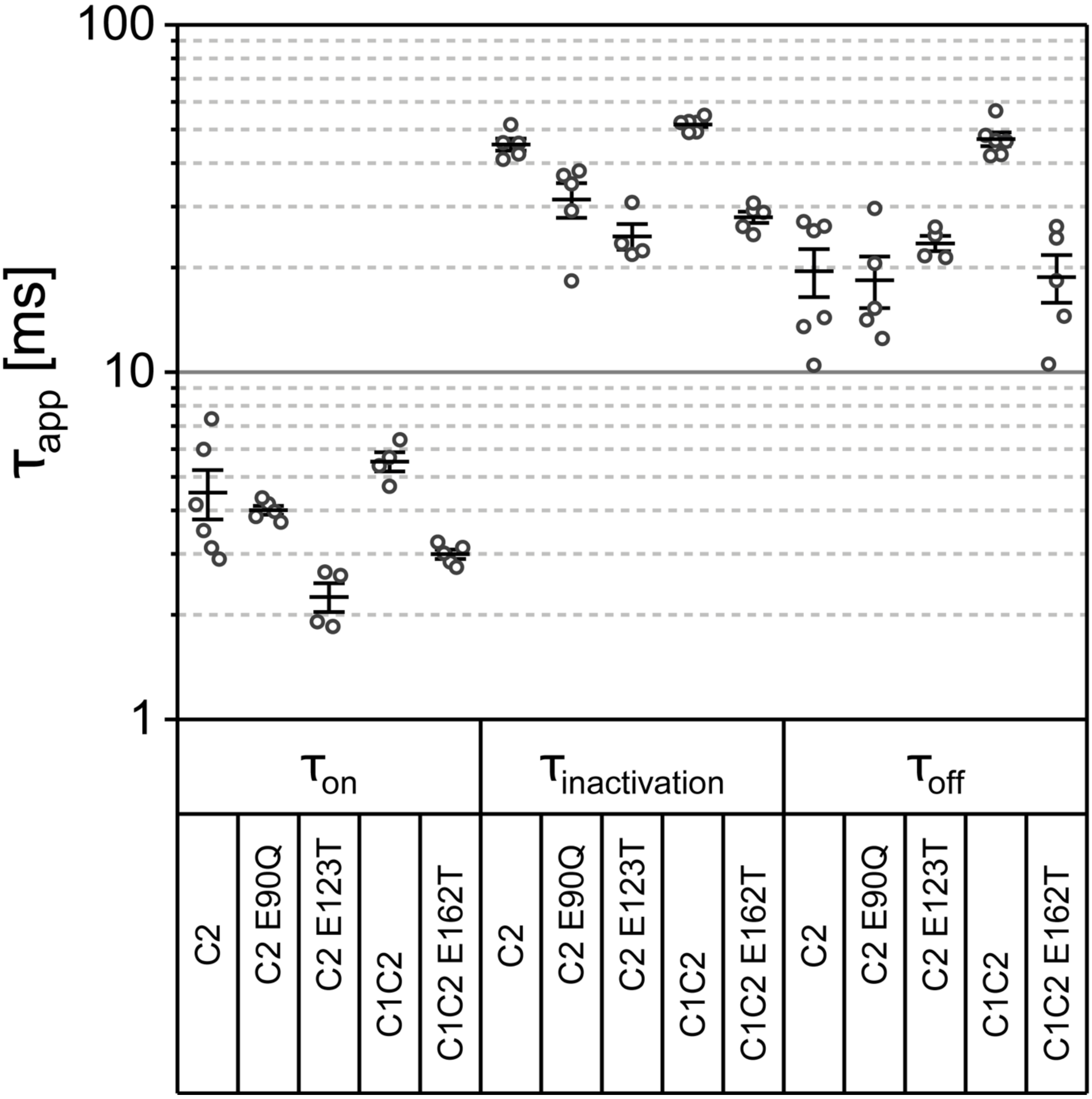

Supplementary Figure 5. Apparent kinetics were obtained via a bi-exponential fit of ND7/23 cell datasets (see SI Fig 1). Channel opening (τ_{on}), inactivation ($\tau_{inactivation}$) and closing (τ_{off}) kinetics extracted at high 140 mM [NaCl]e. Lines and bars represent the mean ± S.E.M and dots represent single measurements. N is a number of biological replicas. For C2: N = 6 for τ_{on} & τ_{in} and N = 5 for τ_{in}; C2-E90Q: N = 5 for all 3 kinetics; C2-E123T: N = 4 for all 3 kinetics; C1C2: N = 4 for τ_{on} and N = 6 for τ_{in} & τ_{off}; C1C1-E162T: N = 5 for all 3 kinetics.

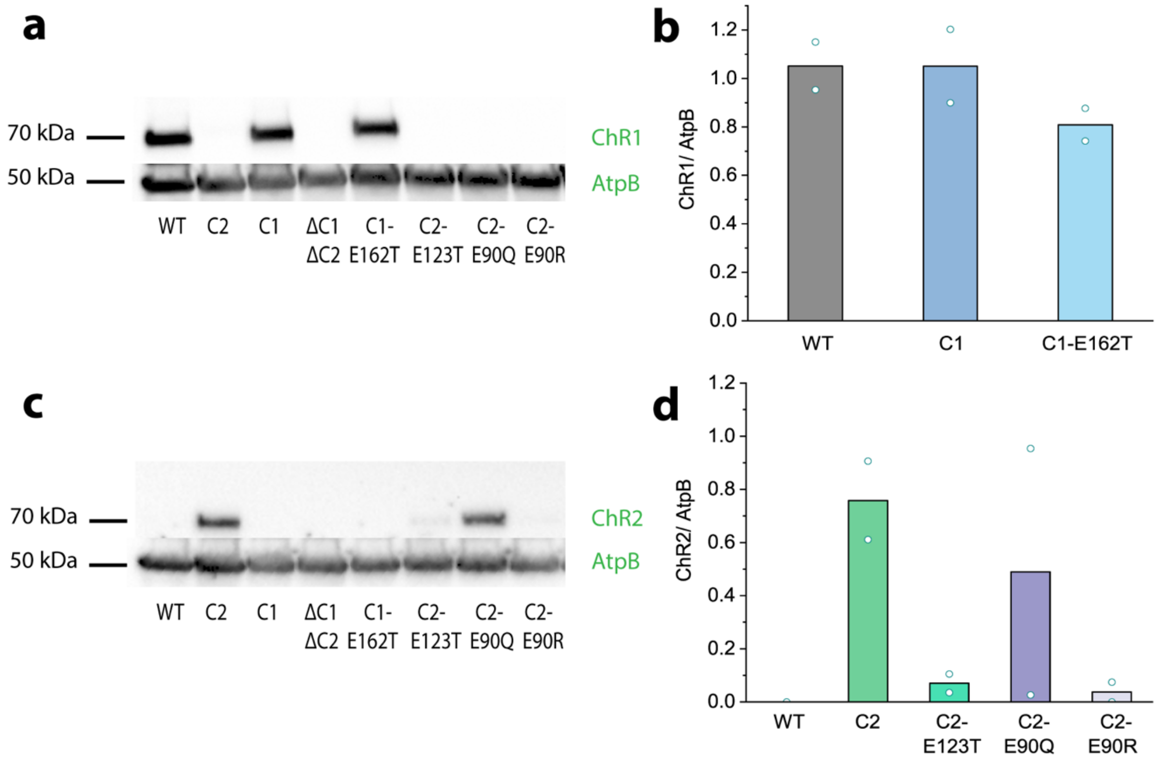

Supplementary Figure 6. Protein immunoblotting evaluation of ChR1 and ChR2 expression levels in vegetative *Chlamydomonas reinhardtii*. Protein immunoblotting of C1 and C2 strain cells, the double knock-out ΔC1ΔC2 and point mutants using anti-ChR1 serum (**a**), anti-ChR2 serum (**c**) and secondary HRP-conjugated antibody for chemiluminescence detection. An ATP synthase subunit B (AtpB) antibody (53 kDa) was used as a loading control. **b** ChR1 expression rates relative to the loading control AtpB. **d** ChR2 expression rates relative to the loading control. Lines represent the mean value. N = 2 independent experiments.

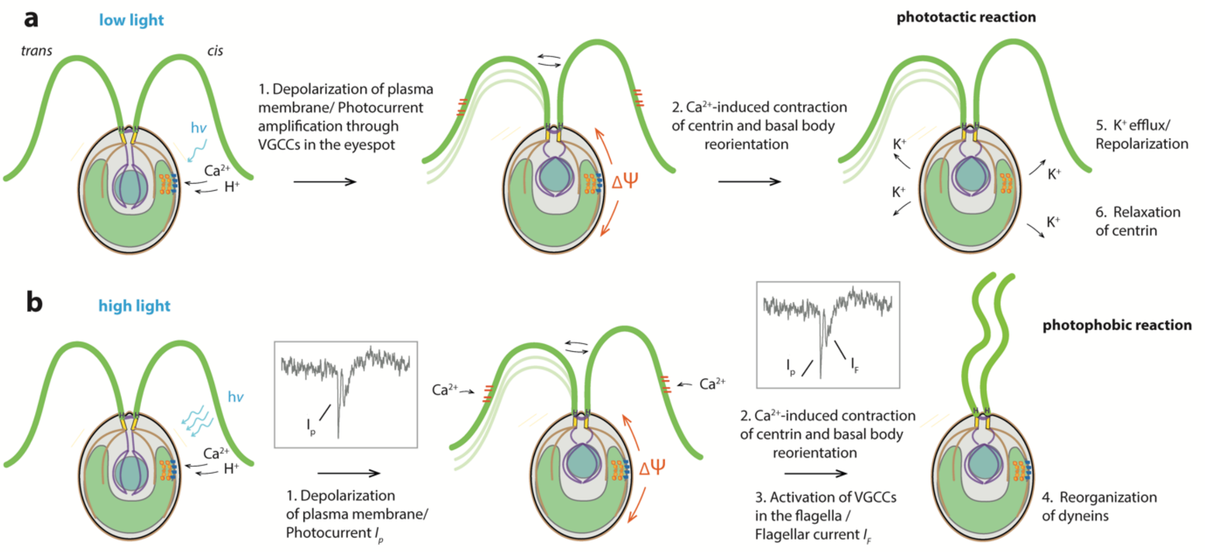

Supplementary Figure 7. Mechanism of a photophobic and phototactic reaction in *Chlamydomonas reinhardtii*. **a** Under low light conditions when less than 1% of ChRs (blue) located in the plasma membrane of the eyespot are excited and the influx of Ca^{2+} and H^+ is low, an amplification of an electrical signal through voltage-gated secondary channels (VGCCs) located in the eyespot are believed to contribute to the depolarization of the plasma membrane (shown as voltage change $\Delta\Psi$)[1]. Meanwhile, Ca^{2+} propagates over microtubule D4 routlets[2] (brown) towards the basal bodies (yellow) and binds to a centrin (violet), protein located in the connecting fiber between two basal bodies, in the filaments that are linking basal bodies to the nucleus (cyan) and in the inner-arm dyneins in the intraflagellar region[3,4]. This leads to a contraction of centrin-based filaments, reduction of the angle between basal bodies, their reorientation and change in the flagellar beating pattern[3,4]. Thus, the *trans* flagellum starts beating with an attenuated amplitude and increased frequency leading to a transient loss of synchrony in a beating pattern of *cis*-*trans* flagella couple[5]. With the K^+ efflux and membrane repolarization[6,7] accompanied by a relaxation of the centrin, a cell changes its swimming direction (phototactic reaction) and restores an initial breast-stroke beating pattern. Since the activation of voltage-gated Ca^{2+} channels (red) in the flagellar region is absent or negligible, no sufficient intraflagellar Ca^{2+} influx occurs to trigger the photophobic reaction[7,8]. **b** Upon bright illumination, when nearly all the ChRs are excited a high influx of Ca^{2+} and H^+ from the extracellular medium into the eyespot region is observed[6–9]. It is followed by a strong depolarization of the plasma membrane[6,7,9] and the subsequent activation of Cav2, voltage-gated Ca^{2+} channels located in the distal part of the flagella[10,11], that initiates a Ca^{2+}-driven flagellar current I_F. Together with the Ca^{2+}-dependent centrin contraction and basal bodies reorientation, the elevation of intraflagellar Ca^{2+} triggers rearrangement of dyneins in a wave-form and a cell starts swimming backwards performing a photophobic reaction. This behavior recruits a complex of Ca^{2+} binding and phosphorylation processes[12–16] with a contribution of motor proteins involved in a

spatial and temporal dynein regulation[17]. Finally, accompanied by the K^+ efflux, membrane repolarization and subsequent relaxation of the centrin, the cell switches its swimming direction (phototactic motion) and embraces the initial breast-stroke mode.

Table 1: Buffers used for I-V recordings on ND7/23 cells.

	**NaCl [mM]**	**KCl [mM]**	**CsCl [mM]**	**$CaCl_2$ [mM]**	**$MgCl_2$ [mM]**	**HEPES/Tris [mM]**	**EDTA [mM]**	**NMGCl [mM]**	**Osmolarity [mOsm]**
External buffers:									
**high $[NaCl]_e$, pH 7.2**	140	1	1	2	2	10	0	0	320
**high $[CaCl_2]_e$, pH 7.2**	1	1	1	70	2	10	0	0	320
**high $[NMGCl]_e$, pH 7.2**	1	1	1	2	2	10	0	140	320
**high $[CaCl_2]_e$, pH 9**	1	1	1	70	2	10	0	0	320
Internal Buffers:									
**high $[NaCl]_i$, pH 7.2**	110	1	1	2	2	10	10	0	290

Supplementary References

1. Kateriya, S., Nagel, G., Bamberg, E. & Hegemann, P. 'Vision' in single-celled algae. *News in Physiological Sciences* **19**, 133–137 (2004).
2. Mittelmeier, T. M., Boyd, J. S., Lamb, M. R. & Dieckmann, C. L. Asymmetric properties of the *Chlamydomonas reinhardtii* cytoskeleton direct rhodopsin photoreceptor localization. *J. Cell Biol.* **193**, 741–753 (2011).
3. Hayashi, M., Yagi, T., Yoshimura, K. & Kamiya, R. Real-time observation of Ca2+-induced basal body reorientation in *Chlamydomonas*. *Cytoskelet.* **41**, 49–56 (1998).
4. McFadden, G. I., Schulze, D., Surek, B., Salisbury, J. L. & Melkonian, M. Basal body reorientation mediated by a Ca2+-modulated contractile protein. *J. Cell Biol.* **105**, 903–912 (1987).
5. Wan K.Y., Leptos K. C. & Goldstein R.E. Log, lack, sync, slip: the many 'phases' of coupled flagella. *J. R. Soc. Interface* **11**, 20131160 (2014).
6. Harz H., Nonnengässer C. & Hegemann P. The photoreceptor current of the green alga *Chlamydomonas*. *Phil. Trans. R. Soc. Lond. B* **338**, 39–52 (1992).
7. Holland, E. M., Braun, F. J., Nonnengässer, C., Harz, H. & Hegemann, P. The nature of rhodopsin-triggered photocurrents in *Chlamydomonas*. I. Kinetics and influence of divalent ions. *Biophys J.* **70**, 924–931 (1996).
8. Nonnengässer C., Holland E.-M., Harz H. & Hegemann P. The nature of rhodopsin-triggered photocurrents in *Chlamydomonas*. II. Influence of monovalent ions. *Biophys J* **70**, 932–938 (1996).
9. Harz H. & Hegemann P. Rhodopsin-regulated calcium currents in *Chlamydomonas*. *Nature* **351**, 489–491 (1991).
10. Fujiu, K., Nakayama, Y., Yanagisawa, A., Sokabe, M. & Yoshimura, K. *Chlamydomonas* CAV2 encodes a voltage-dependent calcium channel required for the flagellar waveform conversion. *Curr Biol.* **19**, 133–139 (2009).

11. Quarmby, L. Ciliary ion channels: Location, location, location. *Curr Biol.* **19**, 158–160 (2009).
12. Yang, P., Diener, D. R., Rosenbaum, J. L. & Sale, W. S. Localization of calmodulin and dynein light chain LC8 in flagellar radial spokes. *J. Cell Biol.* **153**, 1315–1326 (2001).
13. Patel-King, R. S., Benashski, S. E. & King, S. M. A bipartite Ca2+-regulated nucleoside-diphosphate kinase system within the <i<Chlamydomonas</i> flagellum. *J. Biol. Chem.* **277**, 34271–34279 (2002).
14. Wargo, M. J. & Smith, E. F. Asymmetry of the central apparatus defines the location of active microtubule sliding in *Chlamydomonas* flagella. *Proc. Natl. Acad. Sci. USA* **100**, 137–142 (2003).
15. Dymek, E. E. & Smith, E. F. A conserved CaM- and radial spoke-associated complex mediates regulation of flagellar dynein activity. *J. Cell Biol.* **179**, 515–526 (2007).
16. Kamiya, R. & Whitman, G. B. Submicromolar levels of calcium control the balance of beating between the two flagella in demembranated models of *Chlamydomonas*. *J. Cell Biol.* **98**, 97–107 (1984).
17. Riedel-Kruse, I. H., Hilfinger, A., Howard, J. & Jülicher, F. How molecular motors shape the flagellar beat. *HFSP J.* **1**, 192–208 (2007).
