# Supplementary material for: Gating and ion selectivity of Channelrhodopsins are critical for photo-activated orientation of Chlamydomonas as shown by in vivo point mutation
Source: Nat Commun. 2022 Nov 25;13:7253. doi: 10.1038/s41467-022-35018-6 (PMC9700795; doi:10.1038/s41467-022-35018-6)
Supplement: Supplementary file 2 — Reporting Summary [file 41467_2022_35018_MOESM2_ESM.pdf]

nature portfolio

Corresponding author(s): Olga Baidukova

Last updated by author(s): 2.11.2022

Reporting Summary

Nature Portfolio wishes to improve the reproducibility of the work that we publish. This form provides structure for consistency and transparency in reporting. For further information on Nature Portfolio policies, see our Editorial Policies and the Editorial Policy Checklist.

Statistics

For all statistical analyses, confirm that the following items are present in the figure legend, table legend, main text, or Methods section.

n/a	Confirmed	
☐	☒	The exact sample size (n) for each experimental group/condition, given as a discrete number and unit of measurement
☐	☒	A statement on whether measurements were taken from distinct samples or whether the same sample was measured repeatedly
☐	☒	The statistical test(s) used AND whether they are one- or two-sided<br>*Only common tests should be described solely by name; describe more complex techniques in the Methods section.*
☒	☐	A description of all covariates tested
☒	☐	A description of any assumptions or corrections, such as tests of normality and adjustment for multiple comparisons
☐	☒	A full description of the statistical parameters including central tendency (e.g. means) or other basic estimates (e.g. regression coefficient) AND variation (e.g. standard deviation) or associated estimates of uncertainty (e.g. confidence intervals)
☐	☒	For null hypothesis testing, the test statistic (e.g. F, t, r) with confidence intervals, effect sizes, degrees of freedom and P value noted<br>*Give P values as exact values whenever suitable.*
☒	☐	For Bayesian analysis, information on the choice of priors and Markov chain Monte Carlo settings
☒	☐	For hierarchical and complex designs, identification of the appropriate level for tests and full reporting of outcomes
☒	☐	Estimates of effect sizes (e.g. Cohen's d, Pearson's r), indicating how they were calculated

Our web collection on statistics for biologists contains articles on many of the points above.

Software and code

Policy information about availability of computer code

Data collection	Experimental data were collected using following software: electrophysiological data from Chlamydomonas reinhardtii and ND7/23 cells (Clampex 10.4, Molecular Devices, Sunnyvale, CA), Chlamydomonas phototaxis assay data (pClamp9, Molecular Devices, Sunnyvale, CA), Chlamydomonas single cell tracking data (pClamp10, Molecular Devices, Sunnyvale, CA; pco.camware v4.12, Kalheim, Germany) and protein immunoblotting data (ChemieDoc MP system, Bio-Rad, Hercules, USA)
Data analysis	Experimental data were analyzed using following software: electrophysiological data from Chlamydomonas reinhardtii and ND7/23 cells (Clampfit 10.4, Molecular Devices; Origin 2019 and Origin 2017, OriginLab), Chlamydomonas single cell tracking data (plugin TrackMate v4.0.1 & v7.6.1, ImageJ2, doi:10.1016/j.ymeth.2016.09.016; Origin 2019, OriginLab), Chlamydomonas phototaxis assay data (Origin 2019, OriginLab) and protein immunoblotting data (ImageLab 2017, BioRad; Origin 2019, OriginLab)

For manuscripts utilizing custom algorithms or software that are central to the research but not yet described in published literature, software must be made available to editors and reviewers. We strongly encourage code deposition in a community repository (e.g. GitHub). See the Nature Portfolio guidelines for submitting code & software for further information.

Data

Policy information about availability of data

All manuscripts must include a data availability statement. This statement should provide the following information, where applicable:

- Accession codes, unique identifiers, or web links for publicly available datasets
- A description of any restrictions on data availability
- For clinical datasets or third party data, please ensure that the statement adheres to our policy

Data supporting the findings of this manuscript are available from the corresponding authors upon reasonable request.

Human research participants

Policy information about studies involving human research participants and Sex and Gender in Research.

Reporting on sex and gender	n/a
Population characteristics	n/a
Recruitment	n/a
Ethics oversight	n/a

Note that full information on the approval of the study protocol must also be provided in the manuscript.

Field-specific reporting

Please select the one below that is the best fit for your research. If you are not sure, read the appropriate sections before making your selection.

☒ Life sciences ☐ Behavioural & social sciences ☐ Ecological, evolutionary & environmental sciences

For a reference copy of the document with all sections, see nature.com/documents/nr-reporting-summary-flat.pdf

Life sciences study design

All studies must disclose on these points even when the disclosure is negative.

Sample size	No statistical tests were applied to predetermine sample size. Sample sizes were similar to those commonly used in this research field. For ND7/23, see the reference: Broser, M., Spreen, A., Konold, P.E. et al. NeoR, a near-infrared absorbing rhodopsin. Nat Commun 11, 5682 (2020). For Chlamydomonas cells: Harz, H., Hegemann, P. Rhodopsin-regulated calcium currents in Chlamydomonas. Nature 351, 489–491 (1991)
Data exclusions	Electrophysiological data from Chlamydomonas with membrane resistance <30 MOhm were excluded from analysis.
Replication	Electrophysiological recordings from Chlamydomonas and ND7/23 cells, protein immunoblotting imaging as well as phototaxis assay and single cell tracking recordings in Chlamydomonas were repeated (multiple cells from multiple ND7/23 transfection and Chlamydomonas culture batches) and always refer to biological replicates.<br>For ND7/23 cells, constructs were measured on single days. Cells were selected from at least two coverslips and experiments were repeated at least three times. For Chlamydomonas, cells were selected from at least three independent culture batches and experiments were repeated at least three times. All attempts of replication in ND7/23 cells were successful. Replications in Chlamydomonas were highly dependent on seal formation (<30 MOhm).
Randomization	During experiments that required buffer exchange randomization was applied and further automated analysis was used whenever it was possible. In other experiments randomization was also used.
Blinding	Blinding was not applied in order to correctly assign the obtained data to the measured mutants and measuring conditions.

Reporting for specific materials, systems and methods

We require information from authors about some types of materials, experimental systems and methods used in many studies. Here, indicate whether each material, system or method listed is relevant to your study. If you are not sure if a list item applies to your research, read the appropriate section before selecting a response.

Materials & experimental systems

n/a	Involved in the study	
☐	☒	Antibodies
☐	☒	Eukaryotic cell lines
☒	☐	Palaeontology and archaeology
☒	☐	Animals and other organisms
☒	☐	Clinical data
☒	☐	Dual use research of concern

Methods

n/a	Involved in the study	
☒	☐	ChIP-seq
☒	☐	Flow cytometry
☒	☐	MRI-based neuroimaging

Antibodies

Antibodies used	Anti-ChR2 mouse monoclonal primary antibody: PROGEN (Heidelberg, Germany), Cat. No. 651180, 15E2<br>Anti-ChR1 rabbit monoclonal primary antibody: provided by Dr. Suneel Kateriya (School of Biotechnology, Jawaharlal Nehru University, New Delhi, India)<br>Anti-AtpB (Beta subunit of ATP synthase) rabbit polyclonal primary antibody: Agrisera (Vännäs, Sweden), Cat. No. AS05 085<br>Secondary horseradish peroxidase-conjugated ECL anti-mouse IgG (H+L) antibody: Invitrogen (Waltham, USA), Cat. No. 62-6520<br>Secondary horseradish peroxidase-conjugated ECL anti-rabbit IgG (H+L) antibody: Invitrogen (Waltham, USA), Cat. No. 31460
Validation	The primary Anti-ChR2 mouse antibody from PROGEN detects the C-terminus of ChR2. The epitope is located intracellularly between amino acids 290 and 309 of ChR2. More information can be found on the website of the manufacturer: https://www.progen.com/anti-Channelrhodopsin-2-mouse-monoclonal-15E2/610180-1<br>The primary Anti-ChR1 rabbit antibody provided by Dr Suneel Kateriya detects the C-terminus of ChR1. More information on preparation and validation of the antibody can be found in the following publication: Awasthi, M., Ranjan, P., Sharma, K., Veetil, S. K. & Kateriya, S. The trafficking of bacterial type rhodopsins into the Chlamydomonas eyespot and flagella is IFT mediated. Sci Rep 6:34646 (2016) DOI: 10.1038/srep34646<br>The primary Anti-AtpB rabbit antibody from Agrisera is based on a KLH-conjugated synthetic peptide derived from algal (chloroplastic and mitochondrial) sequences of beta subunits of F-type ATP synthases. More information can be found on the manufacturer website: https://www.agrisera.com/en/artiklar/atpb-beta-subunits-of-atp-synthase-global-antibody.html

Eukaryotic cell lines

Policy information about cell lines and Sex and Gender in Research

Cell line source(s)	All the Chlamydomonas reinhardtii mutation cells generated with CRISPR-Cas9 technology are based on the wildtype strain CC125 (mt+). ND7/23 cells were ordered from Sigma-Merck (SKU: 92090903-1VL).
Authentication	Chlamydomonas reinhardtii mutation cell lines were authenticated by PCR screening and NG sequencing. ND7/23 cells: European Collection of Authenticated Cell Cultures (ECACC), No. 92090903. ND cells were authenticated by the vendor before shipment and were always authenticated morphologically before measurements.
Mycoplasma contamination	ND7/23 cells were tested negative for contamination by vendors.
Commonly misidentified lines<br>(See ICLAC register)	No commonly misidentified cell lines were used in the study.
